# A Framework for Cervical Cancer Elimination in Low-and-Middle-Income Countries: A Scoping Review and Roadmap for Interventions and Research Priorities

**DOI:** 10.3389/fpubh.2021.670032

**Published:** 2021-07-01

**Authors:** Michelle B. Shin, Gui Liu, Nelly Mugo, Patricia J. Garcia, Darcy W. Rao, Cara J. Broshkevitch, Linda O. Eckert, Leeya F. Pinder, Judith N. Wasserheit, Ruanne V. Barnabas

**Affiliations:** ^1^School of Nursing, University of Washington, Seattle, WA, United States; ^2^Department of Epidemiology, University of Washington, Seattle, WA, United States; ^3^Department of Global Health, University of Washington, Seattle, WA, United States; ^4^Center for Clinical Research, Kenya Medical Research Institute, Nairobi, Kenya; ^5^School of Public Health, Cayetano Heredia University, Lima, Peru; ^6^Department of Obstetrics and Gynecology, University of Washington, Seattle, WA, United States; ^7^Department of Obstetrics and Gynecology, University of Zambia, Lusaka, Zambia; ^8^Department of Medicine, University of Washington, Seattle, WA, United States; ^9^Vaccine and Infectious Diseases Division, Fred Hutchinson Cancer Research Center, Seattle, WA, United States

**Keywords:** cervical cancer, cervical cancer screening, human papillomavirus vaccination, scoping review, cervical cancer prevention, cervical cancer elimination

## Abstract

The World Health Organization announced an ambitious call for cervical cancer elimination worldwide. With existing prevention and treatment modalities, cervical cancer elimination is now within reach for high-income countries. Despite limited financing and capacity constraints in low-and-middle-income countries (LMICs), prevention and control efforts can be supported through integrated services and new technologies. We conducted this scoping review to outline a roadmap toward cervical cancer elimination in LMICs and highlight evidence-based interventions and research priorities to accelerate cervical cancer elimination. We reviewed and synthesized literature from 2010 to 2020 on primary and secondary cervical cancer prevention strategies. In addition, we conducted expert interviews with gynecologic and infectious disease providers, researchers, and LMIC health officials. Using these data, we developed a logic model to summarize the current state of science and identified evidence gaps and priority research questions for each prevention strategy. The logic model for cervical cancer elimination maps the needs for improved collaboration between policy makers, production and supply, healthcare systems, providers, health workers, and communities. The model articulates responsibilities for stakeholders and visualizes processes to increase access to and coverage of prevention methods. We discuss the challenges of contextual factors and highlight innovation needs. Effective prevention methods include HPV vaccination, screening using visual inspection and HPV testing, and thermocoagulation. However, vaccine coverage remains low in LMICs. New strategies, including single-dose vaccination could enhance impact. Loss to follow-up and treatment delays could be addressed by improved same-day screen-and-treat technologies. We provide a practical framework to guide cervical cancer elimination in LMICs. The scoping review highlights existing and innovative strategies, unmet needs, and collaborations required to achieve elimination across implementation contexts.

## Introduction

Globally, there are more than half a million new cervical cancer cases and more than a quarter-million cervical cancer-related deaths each year (1). Due to effective screening and treatment of precancerous lesions and cancer, high-income countries have seen marked decreases in cervical cancer incidence and mortality in recent decades (2–4). High coverage of human papillomavirus (HPV) vaccination has also contributed to declines in HPV prevalence and cervical lesions in countries with established national vaccination programs (5). Given the success of effective interventions for prevention and treatment, the World Health Organization (WHO) issued a call in 2018 to eliminate cervical cancer as a public health problem globally, defined as an incidence rate <4 per 100,000 women-years (6). To achieve this goal, the WHO proposes an intermediate 90–70–90 target (also known as the “triple-intervention” strategy), which aims to vaccinate 90% of girls by age 15, screen 70% of women with a high-performance test by age 35 and again by 45, and treat 90% of women with cervical disease (6). Australia is projected to achieve elimination by 2050, and other high-income countries are following close behind (7).

However, the timeline for cervical cancer elimination is significantly longer in low-and-middle-income countries (LMICs) (8). This longer timeline is due in part to the current higher burden of disease. More than 80% of cervical cancer cases occur in LMICs (9), where age-standardized cervical cancer mortality rates are at least 6-fold higher than in high-income countries (10). These disparities reflect low access to prevention and treatment due to limited infrastructure, technical expertise, and resources (10). Of the 118 million women who received the HPV vaccine by 2016, only 1.4 million (1%) lived in LMICs (11). Implementation of successful screening programs in LMICs has been challenged by the lack of equipment and personnel to initiate and maintain the screening program, the financial and logistical burden of multiple visits, high rates of loss-to-follow-up, and lack of resource allocation for specialized training (12).

The science, technology, and implementation of cervical cancer prevention and treatment are changing rapidly, with effective and scalable strategies on the horizon. A recent modeling analysis of 78 LMICs demonstrated the importance of successful implementation and scale-up by predicting that the triple-intervention would reduce cervical cancer mortality of women ages 30–69 years by 33.9% (24.4–37.9 per 100,000 women) by 2030 and almost 99% by 2120 (13). The purpose of this scoping review is to (1) synthesize the evidence on the effectiveness of available and emerging cervical cancer elimination strategies, (2) provide a mechanism for visualizing how primary and secondary prevention methods work together using a logic model framework, and (3) highlight gaps in evidence in primary and secondary prevention and propose research priorities to address these gaps and accelerate progress toward elimination.

## Materials and Methods

Our team conducted a scoping review of the literature on primary and secondary cervical cancer prevention strategies using methods adapted from Arksey and O'Malley (14). A scoping review is a technique used to “map” the relevant literature when the field of interest is broad. It differs from a traditional systematic review in that it includes additional study designs as well as randomized trials and other systematic reviews. We chose this method because the topic of cervical cancer elimination in low-resource settings is one that is complex and some aspects of the science (e.g., one-dose HPV vaccine regimen) are still nascent, yet critically important. Our review focuses on primary and secondary prevention to deliver a more in-depth summary on these strategies. The areas of expertise in cervical cancer prevention on our team include: infectious disease specialists (RB, JW, PG, LE), obstetrician gynecologists (LE, LP, NM), LMIC practitioners (PG, NM), epidemiologists (GL, DR), modeling specialists (GL, DR, CB), a gynecologic oncology fellow (LP), and a nurse (MS). The Preferred Reporting Items for Systematic reviews and Meta-Analyses extension for Scoping Reviews (PRISMA-ScR) Checklist is available in Table 1 (15). The scoping review process is described in detail in Table 2.

**Table 1:** Preferred reporting items for systematic reviews and meta-analyses extension for scoping reviews (PRISMA-ScR) checklist (15).

**Section**	**Item**	**PRISMA-ScR checklist item**	**Reported on page #**
**TITLE**			
Title	1	Identify the report as a scoping review.	2
**ABSTRACT**			
Structured summary	2	Provide a structured summary that includes (as applicable): background, objectives, eligibility criteria, sources of evidence, charting methods, results, and conclusions that relate to the review questions and objectives.	2–4
**INTRODUCTION**			
Rationale	3	Describe the rationale for the review in the context of what is already known. Explain why the review questions/objectives lend themselves to a scoping review approach.	2
Objectives	4	Provide an explicit statement of the questions and objectives being addressed with reference to their key elements (e.g., population or participants, concepts, and context) or other relevant key elements used to conceptualize the review questions and/or objectives.	2–4
**METHODS**			
Protocol and registration	5	Indicate whether a review protocol exists; state if and where it can be accessed (e.g., a Web address); and if available, provide registration information, including the registration number.	N/A
Eligibility criteria	6	Specify characteristics of the sources of evidence used as eligibility criteria (e.g., years considered, language, and publication status), and provide a rationale.	Table 2
Information sources^*^	7	Describe all information sources in the search (e.g., databases with dates of coverage and contact with authors to identify additional sources), as well as the date the most recent search was executed.	Table 2
Search	8	Present the full electronic search strategy for at least 1 database, including any limits used, such that it could be repeated.	Supplementary Table 1
Selection of sources of evidence^†^	9	State the process for selecting sources of evidence (i.e., screening and eligibility) included in the scoping review.	2, 4, Table 2
Data charting process^‡^	10	Describe the methods of charting data from the included sources of evidence (e.g., calibrated forms or forms that have been tested by the team before their use, and whether data charting was done independently or in duplicate) and any processes for obtaining and confirming data from investigators.	2, 4, Table 2
Data items	11	List and define all variables for which data were sought and any assumptions and simplifications made.	2, 4, Supplementary Table 2
Critical appraisal of individual sources of evidence^§^	12	If done, provide a rationale for conducting a critical appraisal of included sources of evidence; describe the methods used and how this information was used in any data synthesis (if appropriate).	N/A
Synthesis of results	13	Describe the methods of handling and summarizing the data that were charted.	2, 4, Table 2
**RESULTS**			
Selection of sources of evidence	14	Give numbers of sources of evidence screened, assessed for eligibility, and included in the review, with reasons for exclusions at each stage, ideally using a flow diagram.	Numbers of studies retrieved and included reported in Supplementary Table 1
Characteristics of sources of evidence	15	For each source of evidence, present characteristics for which data were charted and provide the citations.	Supplementary Table 2
Critical appraisal within sources of evidence	16	If done, present data on critical appraisal of included sources of evidence (see item 12).	N/A
Results of individual sources of evidence	17	For each included source of evidence, present the relevant data that were charted that relate to the review questions and objectives.	4–12
Synthesis of results	18	Summarize and/or present the charting results as they relate to the review questions and objectives.	4–12
**DISCUSSION**			
Summary of evidence	19	Summarize the main results (including an overview of concepts, themes, and types of evidence available), link to the review questions and objectives, and consider the relevance to key groups.	12, 13, Table 6
Limitations	20	Discuss the limitations of the scoping review process.	13
Conclusions	21	Provide a general interpretation of the results with respect to the review questions and objectives, as well as potential implications and/or next steps.	13, 14
**FUNDING**			
Funding	22	Describe sources of funding for the included sources of evidence, as well as sources of funding for the scoping review. Describe the role of the funders of the scoping review.	14, 20

*JBI, Joanna Briggs Institute; PRISMA-ScR, preferred reporting items for systematic reviews and meta-analyses extension for scoping reviews*.

* *Where sources of evidence (see second footnote) are compiled from, such as bibliographic databases, social media platforms, and Web sites*.

† *A more inclusive/heterogeneous term used to account for the different types of evidence or data sources (e.g., quantitative and/or qualitative research, expert opinion, and policy documents) that may be eligible in a scoping review as opposed to only studies. This is not to be confused with information sources (see first footnote)*.

‡ *The frameworks by Arksey and O'Malley (6) and Levac et al. (7) and the JBI guidance (4, 5) refer to the process of data extraction in a scoping review as data charting*.

§ *The process of systematically examining research evidence to assess its validity, results, and relevance before using it to inform a decision. This term is used for items 12 and 19 instead of “risk of bias” (which is more applicable to systematic reviews of interventions) to include and acknowledge the various sources of evidence that may be used in a scoping review (e.g., quantitative and/or qualitative research, expert opinion, and policy document)*.

**Table 2:** Scoping review framework and description of methods.

**Arksey and O'Malley framework stage**	
1. Identifying the research question	• What do we already know? • What are the gaps in evidence? • What are the relevant innovations? • What are the most pressing questions we need to answer to scale-up cervical cancer elimination strategies?
2. Identifying relevant studies	• Search sources: PubMed, Scopus, reference lists, and governmental and non-profit organizational websites • Inclusion criteria: ◦ Programmatic interventions identified by the WHO life course model (16) ◦ English language published between 2010 and 2020 ◦ Peer-reviewed studies and conference abstracts that examined efficacy, effectiveness, sensitivity, and/or specificity of existing and emerging strategies to prevent HPV infection and detect or treat cervical precancers and cervical cancer ◦ Interventional studies that address innovations and implementation gaps
3. Study selection	• Systematic reviews, meta-analysis and randomized controlled trials were prioritized for each intervention. When these were not available, we selected longitudinal and prospective cohort studies with relative risks or odds ratios that address HPV acquisition, progression to precancer and treatment of cancer. Individual cross-sectional studies were reviewed only if sufficient data from the above types of studies were not available.
4. Charting the data	Two authors (MS and GL) screened the search results for relevant articles and independently extracted data relevant to the key questions. The last update of the search was conducted in August 2020, and the following data was extracted using Microsoft Excel sheet (see Supplementary Material): • Primary prevention: author, year, study design, location, population, exposure, unit of exposure, comparison, comparison number of doses, outcomes, sample size, key findings • Secondary prevention: author, year, intervention, study design, location, population, intervention, comparison, outcomes, clinical endpoint, key findings
5. Collating, summarizing, and reporting the results	• As specified by Arksey and O'Malley, a narrative literature review method was used, in which data synthesis and interpretation of the findings were conducted simultaneously, in an iterative manner with the research team. In addition to the narrative synthesis, we followed the Centers for Disease Control and Prevention's Program Evaluation Framework to organize the evidence on the available and emerging strategies for cervical cancer elimination into a logic model (17).

We chose to review articles published beginning in year 2010, because while the HPV vaccine was introduced to the world in 2006, it was not until 2010 that it became incorporated into the national immunization program in LMIC settings, starting with Bhutan in 2010 and Rwanda in 2011 (18). We used the WHO's “Life-course approach to cervical cancer interventions” as a guide to organize our review (6). We defined primary prevention as the prevention of HPV infection and secondary prevention as the detection and treatment of precancerous cervical lesions.

Two authors (MS and GL) screened the search results for relevant articles and extracted data independently. All authors provided feedback on study selection, data extraction, and synthesis, which informed further search and interpretation of the findings. Our search for primary prevention strategies focused on the effect of HPV vaccination, voluntary medical male circumcision (VMMC), tobacco cessation, condom use as mentioned by the WHO (16), and vaginal dysbiosis. While vaginal dysbiosis is not commonly mentioned as a risk factor for cervical cancer, we decided to include it in our review because there is relatively strong and consistent evidence that the vaginal microbiota play a role in cervical cancer pathology (19–22).

We divided secondary prevention into screening and treatment strategies. For screening, we compared cytology, HPV tests, and visual inspection with acetic acid (VIA). As the WHO recommends screening with a high-performance test equivalent to or better than HPV testing, we reviewed implementation challenges of HPV testing in low-resource settings. We recognize HPV-based screening may be considered a primary prevention strategy as it can detect individuals in whom cervical cancer has not occurred. However, for the purpose of our review and following the WHO guidelines, we reviewed this screening method under secondary prevention. For immediate treatment of precancerous lesions, we focused on cryotherapy, thermal ablation, and loop electrosurgical excision procedure (LEEP). We identified emerging screening and triage options in LMICs as HPV self-sampling, oncogenesis biomarkers, optical techniques, such as portable colposcopes and automated visual evaluation, and therapeutic vaccines. We also dedicated a section to prevention of cervical cancer among women living with HIV to highlight the differences in HPV acquisition and progression in this population.

## Results

### The Logic Model

**Figure 1:**
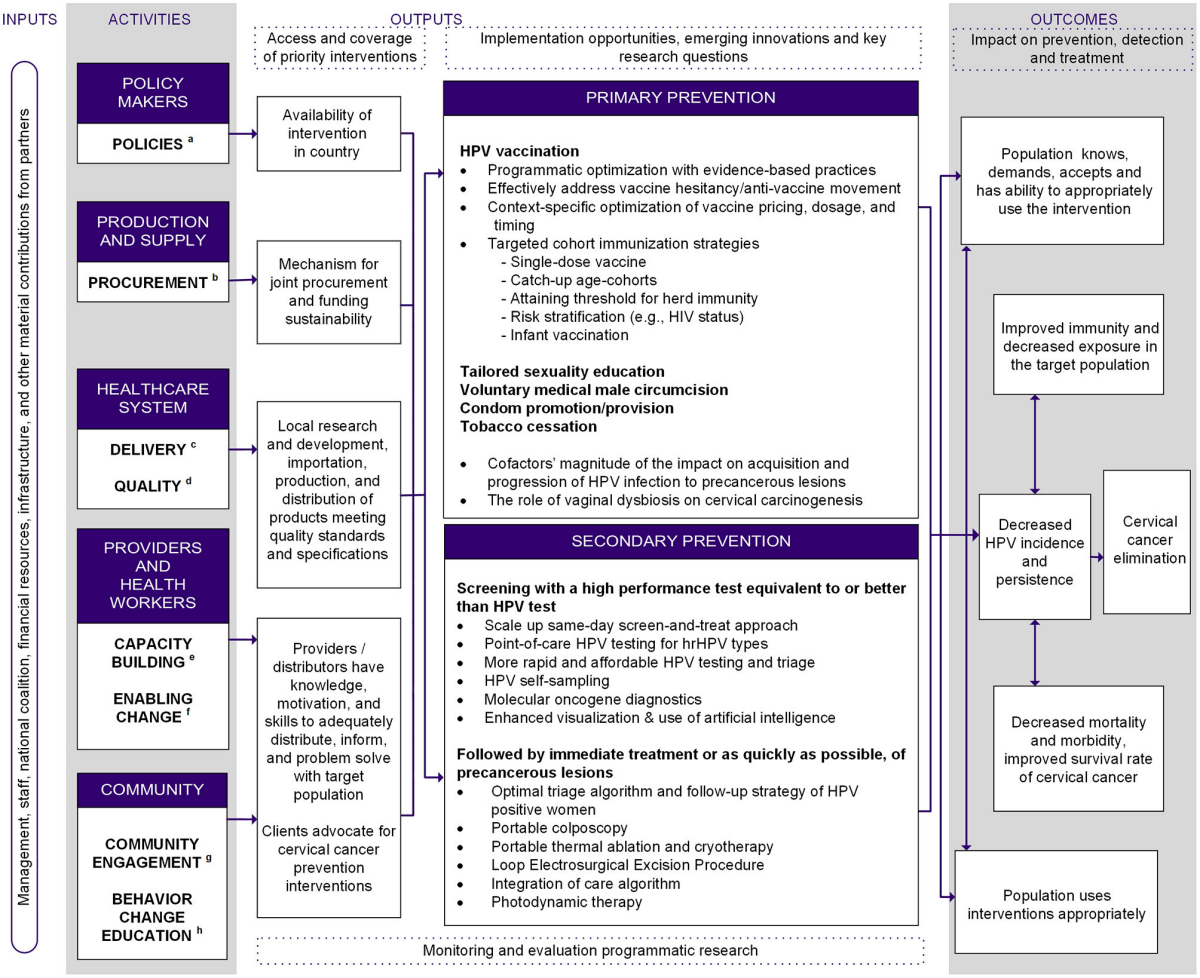
Logic model for comprehensive, intersectoral cervical cancer prevention. ^a^Policies: develop and implement policies, legislation regulations, and registrations. ^b^Procurement: develop and implement provision, production, procurement and training strategies. ^c^Delivery: develop and implement delivery system, strategy for management, training, and maintain motivation among providers and distributors. ^d^Quality: develop and implement an external and internal quality control system. ^e^Capacity building: develop provider and health worker skills training, infrastructure and capacity building. ^f^Enabling change: cultivate dialogue to promote adoption of innovative technologies and approaches (e.g., task-shifting/sharing) to simplify care delivery and break conflicts of interest. ^g^Community engagement: demand promotion by empowering local stakeholders and advocacy. ^h^Behavior change education: develop and implement intervention strategy for information, education, and communication for behavior change.

The logic model summarizes and describes the process flow for cervical cancer elimination (Figure 1). Step one is marshaling crucial resources needed for sustainable cervical cancer elimination programs in LMICs: healthcare worker capacity, political commitment, funding, and infrastructure and material support from domestic and global partners (“Inputs”) as explicitly captured in the WHO's “Global strategy toward eliminating cervical cancer as a public health problem” (16). To acquire these resources, key stakeholders must build capacity within their sectors and foster cross-sector collaborations (“Activities”). The intended impact of the program is organized into primary and secondary prevention nodes (“Outputs”). For each prevention strategy, we summarized what is known, the impact of the intervention, and innovations under development. All strategies lead to “Outcomes,” which are the expected intermediate impacts on the path toward cervical cancer elimination. Synthesizing the efficacy of current interventions identified gaps in innovation in primary and secondary prevention, which are summarized in Figure 1.

### Primary Prevention

#### Current State of Science

##### HPV Vaccines

HPV vaccines, which have the potential to prevent 90% of cervical cancer cases (6), are by far the most efficacious primary prevention modality. Among HPV-naïve adolescent girls and young women, the efficacy of available vaccines (bivalent, quadrivalent, and nonavalent) is >95% for preventing HPV infections and cervical lesions caused by vaccine-targeted HPV types (23–28). Since the median age of sexual initiation is 15–17 in many populations (29), the WHO recommends vaccination programs to target 9–13 year-old girls (30). Mathematical modeling analyses from 73 LMICs found that routine vaccination at age 9 and multi-cohort vaccination of girls ages 10–14 reduced cervical cancer deaths by 30–40%, or 1.2–1.8 million, over the lifetime of the vaccinated cohorts in addition to the number of deaths averted with routine vaccination only (31). Unfortunately, as of 2018, only 13 of the 90 countries who had introduced HPV vaccine into their national programs were lower income countries (32). A comprehensive strategy is needed while key interventions, such as HPV vaccination is scaled-up (6).

Further, the cost-effectiveness of vaccination strategies is dependent on the vaccine price, which is likely to drop significantly as new vaccines being developed in India and China increase HPV vaccine supply over the next ten years (33). The economic landscape of HPV vaccines is quickly changing to promote access to the vaccines in LMICs and adoption of national HPV immunization programs. Just prior to the Global Vaccine Summit 2020, five manufacturers of HPV vaccines committed to increasing the supply in Gavi-supported countries (34). The proportion of LMICs with national programs is low (22 of the 78 LMICs vs. 50 of the 57 high-income countries as of 2020) (35, 36), but increasing, since the Gavi Alliance negotiated the price of the vaccine as low as $4.50 USD per dose for the poorest countries in 2013 (37). Economic evaluations from modeling studies suggest that HPV vaccination of adolescent girls is cost-effective for most countries, especially low-income countries (38, 39) and when the vaccine price is affordable for the country's income level (40).

##### Voluntary Medical Male Circumcision

VMMC for HIV-negative men has been shown to reduce penile HPV viral load of incident infections and the persistence of prevalent HPV infections, which likely reduces male-to-female HPV transmission (41, 42). Even when transmission occurs, female partners of circumcised men had lower HPV viral load (42). The incidence of high-risk HPV (hrHPV) infection was lower among women whose male partners received circumcision than those who did not (incidence rate ratio = 0.77, 95% CI: 0.63–0.93) (43).

##### Other Cofactors

HPV incidence is inversely associated with the frequency of condom use (44). In a longitudinal study with 8 months of follow-up, female college students who reported using condoms during all vaginal intercourse were 70% less likely to acquire a new infection than those who reported using condoms <5% of the time, after adjusting for the number of new partners and estimated number of previous partners of the male partner (45).

Current smokers were 1.6 times more likely than never smokers to have prevalent hrHPV infection than never smokers (95% CI: 1.2–2.1) and 1.4 times more likely to have newly detected hrHPV infection (95% CI: 1.0 −1.9) (46). This increased risk may be explained by the lack of immune response after a natural infection among people who smoke, which lowers their defense against subsequent infection (46).

Vaginal dysbiosis, including but not limited to bacterial vaginosis, is positively correlated with prevalent HPV infection and cervical intraepithelial neoplasia (CIN) among women with and without HIV (20, 47–49). While vaginal dysbiosis increased risk of persistent HPV infection (50), high grade squamous intraepithelial lesions (HSIL) and cervical cancer (51), a healthy cervicovaginal microbiome, dominated by multiple species of Lactobacillus bacteria, was associated with lower prevalence of hrHPV infection (52). There is a need to further investigate the complex relationship between the microbiome of the female reproductive tract, HPV and cervical carcinogenesis (53).

Table 3 summarizes the efficacies and effect of co-factors in prevention of HPV infection. The clinical endpoints and the intervention effect are not uniform; hence, the potential impact of different preventative strategies cannot be compared to each other. However, this is precisely an evidence gap that can hinder implementation and evaluation prevention efforts. Lastly, the co-factors mentioned in this table do not represent an exhaustive list of risk factors for cervical cancer or HPV infection.

**Table 3:** Summary of HPV vaccine efficacy and effect of co-factors on HPV-related clinical endpoints.

**Primary prevention method**	**Endpoint**	**Effect (%, 95% CI)**	**References**
**HPV VACCINE** ^*^			
(HPV)-16/18 AS04-adjuvanted vaccine	CIN2-3 associated with HPV 16/18 [mean follow-up (f/u): 34.9 months]	92.9 (96.1% CI: 79.9–98.3)	(23)
Quadrivalent vaccine (HPV 6, 11, 16, 18)	CIN1-3 or adenocarcinoma *in situ* associated with HPV 6, 11, 16, 18 (mean f/u: 36 months)	100.0 (95% CI: 94.0–100.0)	(25)
	CIN 2 or 3, adenocarcinoma *in situ*, or cervical cancer related to HPV 16 or 18 (mean f/u: 36 months)	98.0 (95.89% CI: 86.0–100.0)	(26)
Nonavalent vaccine (HPV 6, 11, 16, 18, 31, 33, 45, 52, and 58)^†^	CIN 2 or 3, adenocarcinoma *in situ*, invasive cervical carcinoma, and vulvar disease related to HPV 31, 33, 45, 52, and 58 (up to 6 years)	97.4 (95% CI: 85.0–99.9)	(27)
	High-grade cervical, vulvar, or vaginal disease related to HPV-31, 33, 45, 52, and 58 (up to 54 months)	96.7 (95% CI: 80.9–99.8)	(28)
**VOLUNTARY MEDICAL MALE CIRCUMCISION**			
	HPV prevalence risk ratios among women partners 24 months after intervention	0.72 (95% CI: 0.60–0.85)	(43)
	Incidence rate ratio of hrHPV^‡^	0.77 (95% CI: 0.63–0.93)	(43)
**TOBACCO USE** ^**§**^			
	Odds ratio of hrHPV infection at baseline	1.60 (95% CI: 1.20–2.10)	(46)
	Odds ratio of CIN2-3 at baseline	1.80 (95% CI: 1.30–2.50)	(46)
	Risk ratio of incident hrHPV infection	1.40 (95% CI: 1.00–1.90)	(46)
	Risk ratio of incident CIN2-3	3.60 (95% CI: 1.50–8.60)	(46)
**CONDOM USE** ^**¶**^			
	Percent reduction of incident genital HPV infection	70.0% (95% CI: 40.0–90.0)	(45)
**VAGINAL DYSBIOSIS**			
	Risk ratio of incident HPV infection	1.35 (95% CI: 1.18–1.50)	(19)
	Risk ratio of HPV persistence	1.14 (95% CI: 1.01–1.28)	(19)
	Risk ratio of high grade squamous intraepithelial lesion/squamous cell changes	2.01 (95% CI: 1.40–3.01)	(19)

* *Indicates vaccine efficacy*.

† *The results for HPV 6, 11, 16 and 18 not included in the table because of wide confidence intervals containing 0*.

‡ *HPV 6, 18, 31, 33, 35, 39, 45, 51, 52, 56, 58, 59, 66, and 68*.

§ *Comparing current smokers to never smokers*.

¶ *Comparing condom use 100% of the time during 8 months to those whose partners used condoms <5% of the time among women who had never had vaginal intercourse or had first had intercourse with one male partner within the previous 3 months of the study*.

#### Priority Research Questions for Primary Prevention Strategies

We prioritized implementation and scale-up of HPV vaccination in primary prevention opportunities, as it directly addresses the known causal agent of most cervical cancer. Several barriers specific to LMICs have been identified, such as costs associated with the vaccine and service delivery, political commitment, challenges specific to the target population of adolescent girls, and global vaccine shortage (36). In addition, the scale-up of HPV vaccination programs has faced challenges of vaccine hesitancy due to concerns about the safety and side effects of the vaccine and the belief that vaccination can lead to sexual promiscuity (54).

Successful implementation and scale-up of HPV vaccine programs depends on achieving and maintaining high rates of vaccine uptake (5), which requires context-informed delivery strategies. Microplanning for HPV vaccination encompasses logistical considerations, such as delivery of multi-dose vaccines, reaching out-of-school girls, and alignment with the school calendar, as well as careful navigation of complex sociopolitical settings where sensitization to reproductive health can be delicate (55). For example, Australia, which has one of the highest coverages in the world (as of 2007, 70% of 12–17-year-old girls nationwide completely vaccinated with the 3-dose regimen), attributes its success to the publicly funded school-based HPV immunization program (56). The high enrollment rate in schools serves as a key facilitator of high coverage, as 98.5% of girls aged 14 are enrolled in schools in Australia (56). In contrast, an estimated 18.6 million girls (23%) aged 6–11 years old are not enrolled in primary schools in sub-Saharan Africa (57). Gavi-eligible countries have proposed many strategies to locate hard-to-reach girls, such as enumeration by community health workers and mapping health facilities (55), but more research is needed to evaluate the currently existing programs and develop reproducible, validated interventions (58).

HPV vaccine introduction efforts have faced more challenges than the pneumococcal conjugate, rotavirus and inactivated polio vaccines, which can be readily integrated into existing infant immunization schedules. While low school attendance in some settings limits school-based routine vaccination, most LMICs have well-established infant immunization programs (59, 60). The competition for resources to scale-up HPV vaccine programs will likely worsen in LMICs due to the COVID-19 pandemic (61), as prevention activities have been disrupted (62), and vaccine coverage is expected to decrease even in high-income countries (63). Development of an HPV vaccine that can be safely administered to infants and maintain immunity into adulthood like the Hepatitis B vaccine would bypass some of the current logistical and financial challenges and expand access and coverage.

Immune correlates of protection against HPV are poorly understood (64, 65), preventing optimization of vaccine dosing schedules, such as through multi-cohort vaccination or reduced-dosing strategies (66, 67). A better understanding of serological correlates of protection can help define the vaccine dosage needed for protection (65). In particular, reduced-dose vaccination, either with a single dose or extended interval dosing (at least 12 months between the first and the second dose) (66), would have significant programmatic implications and allow for increased vaccination coverage in settings with limited healthcare access, infrastructure, personnel, and financial resources (68, 69). Among women who did not complete the required number of HPV vaccine doses, one dose induced robust immune responses with comparable decreases in precancerous lesions compared to women who received multiple doses (70, 71). Clinical trials to evaluate single-dose HPV vaccine efficacy and the duration of protection are underway to inform decisions about adoption of a one-dose regimen (see NCT03675256, NCT03747770, NCT03728881 on ClinicalTrials.gov) (72).

For all scenarios of expanded HPV vaccine coverage, clear communication addressing vaccine hesitancy and increasing parents', caregivers', and adolescents' acceptance of an HPV vaccine are critical. Unfortunately, literature on vaccine hesitancy from LMICs is scarce (73). In their 2014 report, the WHO's Strategic Advisory Group of Experts on Immunization defined vaccine hesitancy as “delay in acceptance or refusal of vaccination despite availability of vaccination services” and posited that it is a “complex and context specific” phenomenon (74). The 2018 Wellcome Global Monitor showed that 95% and 92% of those in South Asia and East Africa perceived the vaccine to be safe, compared to the 59% of the participants in Western Europe (75). As for all vaccines, there needs to be a continued, concerted effort to build trust and send consistent messages about the safety and effectiveness of the HPV vaccines, especially for children (76).

According to the WHO/United Nations International Children's Emergency Fund's joint reporting from 2015 to 2017, factors related to religion, culture, gender, and socioeconomic status were becoming more prominent in vaccine hesitancy in low-income countries, and risk-benefit (scientific evidence) factors in lower-middle-income countries (77). After the launch of a national HPV immunization program in 2012, Colombia reached a first dose coverage level of 94.8% among the target population (girls aged 9–17) by 2014 (78). However, crisis ensued in 2014, during which over 500 girls from a town called Carmen de Bolivar who had received the HPV vaccine months before reported adverse symptoms, such as headache, shortness of breath, and fainting, which was covered widely by the media (79). Although epidemiologic evaluation found no association between the HPV vaccine and the adverse symptoms, public confidence in the vaccine decreased and led to discontinuation of school-based programs (78, 80). The 2016 national coverage rates fell to as low as 14 and 5% for the first and second dose, respectively (79). However, with the help of projects geared toward restoring confidence in HPV vaccination, such as a roundtable of stakeholders and experts including the Ministry of Health and universities, and a communication intervention targeting communities with the highest cervical cancer mortality rates, first dose coverage rose to 34% in 2019 (78). Additionally, media coverage of unconfirmed reports of adverse events in Japan has led their HPV vaccination rate among adolescents born in and after 2002 to drop from about 70% in 2013 to 1% or less in 2019 (81). Experiences in Colombia and Japan demonstrate the importance of community acceptance of the HPV vaccine for achieving and maintaining high coverage. In summary, the priority research questions for primary prevention strategies are:

- Do voluntary male circumcision, condoms, smoking cessation, and treatment for bacterial vaginosis reduce persistent HPV infection, and to what extent?
- How can monitoring and evaluation provide evidence to support best practices for HPV vaccination programs?
- How can we address vaccine hesitancy and the anti-vaccination movement to increase HPV vaccine coverage?
- What are the safety, efficacy, durability, and acceptability of an HPV vaccine administered in infancy?
- What are the efficacy and durability of one-dose HPV vaccination in routine and catch-up vaccination?
- How can we obtain LMIC context-specific estimates of the impact of prevention strategies and make them more reliable and approachable for policy makers?

### Secondary Prevention

#### Current State of Science

##### Screening of Precancerous Lesions

The WHO's cervical cancer elimination targets are to screen 70% of women with a high-performance test by age 35, and again by age 45 (6). Self-reported lifetime prevalence of cervical cancer screening among women in 55 LMICs was only 43.6%, ranging widely from 0.3 to 97.4% (82). A modeling analysis of LMICs predicted that one lifetime screen could lead to elimination in 96% of LMICs by the end of the century, compared to elimination in 60% of LMICs with HPV vaccination only (8).

Screening for cervical cancer is currently conducted by cytology (Papanicolaou/Pap smear screening), HPV DNA testing, or VIA. VIA is currently the predominant cervical cancer screening method in many LMICs due to its relatively low cost and ease of execution compared to cytology or HPV testing (83). VIA can facilitate same-day screen-and-treat algorithms that minimize loss-to-follow-up when coupled with treatment of detected lesions (84). However, the performance of VIA in detecting HSIL or CIN2-3 varies widely (Table 4), as the interpretation of the results is subjective (92). Quality assurance is another challenge, as the number of micro-invasive cancers that are undetected by the screen-and-treat method of VIA and cryotherapy is unknown (92). About 90% of women who screen positive with VIA and receive treatment are unlikely to have pre-malignant cervical lesions, which raises concern for overtreatment (90, 93–95).

**Table 4:** Summary of sensitivity and specificity of cervical cancer screening methods for detecting CIN2-3.

**Sensitivity (%, 95% CI)**	**Specificity (%, 95% CI)**	**References**
**CYTOLOGY**		
65.9 (54.9–75.3)^*^	96.3 (94.7–97.4)	(85)
75.5 (66.6–82.7)^†^	91.9 (88.4–94.3)	(86)
**HPV DNA TESTING**		
88.1(81.4–92.7)	83.7 (74.9–89.8)	(87)
88.3 (73.1–95.5)	73.9 (50.7–88.7)	(87)
94.0 (89.0–97.0)	88.0 (84.0–92.0)	(88)
**VISUAL INSPECTION WITH ACETIC ACID**		
79.2	84.7	(89)
82.4 (76.3–87.3)	87.4 (77.1–93.4)	(90)
78.0 (73.0–83.0)	88.0 (85.0–91.0)	(91)
69.0 (54.0–81.0)	87.0 (79.0–92.0)	(91)
**VISUAL INSPECTION WITH ACETIC ACID AND LUGOL'S IODINE**		
89.0	85.0	(90)
90.0 (85.0–94.0)	83.0 (79.0–86.0)	(91)

* *Conventional Pap*.

† *Liquid-based cytology*.

*Endpoints are all CIN2-3, reference standard = colposcopy with or without biopsy*.

While cytology-based screening is used widely in high-income settings, it requires established healthcare infrastructure, repeat client visits to diagnose precancer, training of pathologists and clinicians, and a robust quality assurance program. Cytology-based screening also has lower sensitivity and specificity compared to HPV DNA testing (12). For these reasons, cytology-based screening is not recommended for scale-up of cervical cancer screening in LMICs (96).

The wide range of sensitivity and specificity of subjective tests, such as cytology and VIA leads to variation in the number of women referred to triaged for treatment. For example, estimates of sensitivity to detect CIN2-3 varied between 40.7 and 73.7% using cervical cytology and 21.9–73.6% using VIA in one study conducted in India, Nicaragua and Uganda (97). Variation in the number of women referred to triage for treatment can have significant implications for the risk of overtreatment and for already-overburdened health systems in low-resource settings.

HPV DNA testing has superior sensitivity compared to cytology and VIA in detecting CIN2-3 (Table 4) and has been used to either replaced cytology as a primary screen or been offered as a co-test (98, 99). The test has a high negative predictive value for detecting CIN2-3, which has the advantage of elongating the screening interval to 5 years for screen-negative women compared to 3 years for cytology (6). However, it also has a low specificity and positive predictive value, which can lead to overtreatment especially among younger women (100).

Large-scale randomized controlled trials have demonstrated the efficacy of HPV testing in reducing cervical cancer incidence, and that HPV testing provides 60–70% greater protection against invasive cervical cancer than cytology (101). Despite also demonstrating cost-effectiveness compared to cytology in multiple settings (102–104), HPV testing has been difficult to scale-up in LMICs due to cost (12). However, there have recently been several successful demonstration projects, as well as the launch and the scale-up of national HPV testing projects in Latin America using careHPV (105). Notably, the Ministry of Health of El Salvador has updated its guidelines to recommend HPV testing (106), Guatemala and Honduras are planning to use HPV testing for cervical cancer screening after demonstration projects (105), and Argentina and Mexico have been offering HPV testing in their public health systems for several years (107).

The WHO endorses self-sampled HPV tests as an additional approach to cervical cancer screening for individuals aged 30–60 years (108). In a meta-analysis, sensitivity or specificity for detecting CIN2-3 was not statistically different between self-sampled and clinician-sampled HPV tests based on polymerase chain reaction (PCR) assay (109). However, self-collected hrHPV assays based on signal amplification (careHPV) had lower sensitivity (pooled ratio: 0.85, 95% CI: 0.80–0.89) and lower specificity (pooled ratio: 0.96, 95% CI: 0.93–0.98) to exclude CIN2-3 compared to the clinician-collected samples. Any potential loss in sensitivity is likely outweighed by increased screening uptake (110). Both self-sampled and clinician-sampled HPV tests with a PCR assay have better sensitivity and can allow for longer screening intervals than cytology-based screening programs. For example, a 15-year cohort study in rural China found that while HPV testing with self-sampling was less sensitive than physician-sampling, it performed equally on screening efficiency and predicting cumulative cases, and was as sensitive as high-quality cytology in detecting cumulative CIN2-3 cases (111).

Self-sampling as an alternative method of screening can overcome barriers, such as access to the health facility and fear associated with pelvic examination, opening new possibilities of reaching underscreened women (112). Acceptability of self-sampling has been reported across cultures and resource-settings (113–117). HPV self-sampling has the potential to make HPV testing more affordable and cost-effective by increasing screening coverage (118). One meta-analysis found women who were offered the option of self-sampling were twice as likely to participate in cervical cancer screening services than those who were offered cytology, VIA, or clinician-collected HPV testing (119). The difference was greater when the sampling kits were sent directly to women's homes or offered door-to-door by a health worker (119). Self-sampling has been incorporated into the national screening guidelines in high income countries, such as the Netherlands, Australia, and Finland (112). Among LMICs, self-sampling was successfully scaled-up in Jujuy province in Argentina (120). Additional research is needed on best practices for self-sampling follow up, such as community collection of specimens, delivery of results, and linkage to treatment.

##### Treatment of Precancerous Lesions

The international consensus is to treat CIN2-3/HSIL by ablation or excision except during pregnancy (121). Cryotherapy and thermal ablation (the latter also known as cold coagulation or thermocoagulation) are the two most commonly used ablative treatments, and LEEP and cold knife conization are commonly used excisional treatments. Meta-analyses that compared the efficacy of cryotherapy, thermal ablation, LEEP, and cold knife conization are summarized in Table 5.

**Table 5:** Summary of efficacy of LEEP, cold knife conization, cryotherapy, and thermal ablation.

**Method**	**Endpoint**	**Pooled cure proportions (%, 95% CI)**	**References**
**LEEP**			
	CIN2-3 negative after 12 months	94.7 (96.3–93.1)	(122)
	HSIL negative after 12 months follow up	92.0 (N/A)	(123)
**COLD KNIFE CONIZATION**			
	CIN2-3 negative after 12 months	98.6 (99.2–98.0)	(124)
**CRYOTHERAPY**			
	CIN2-3 negative after 6 months in LMICs	82.6 (77.4–87.3)	(125)
	CIN2-3 negative at follow up (duration unspecified)	86.0 (83.0–89.0)	(126)
	CIN2-3 negative after 12 months	94.7 (96.1–93.2)	(124)
	HSIL negative after 12 months follow up	80.9 (N/A)	(123)
**THERMAL ABLATION**			
	CIN2-3 negative after 6+ months	91.6 (88.2–94.5)	(125)
	CIN2-3 negative after 6+ months (LMICs only)	82.4 (75.4–88.6)	(125)
	CIN2-3 negative after 4–6 months	93.6 (90.8–96.0)	(127)

The cervical lesion must be small enough to be covered by the equipment and fully visible with no extension into the endocervix or onto the vaginal wall to be eligible for ablative therapy (121, 128). An estimated 50–80% of cervical lesions found during screening are eligible for ablative therapy (128). In meta-analyses, 81–95% of the women treated with cryotherapy were negative for CIN2-3 12 months after treatment (Table 5) (125, 129). Similarly, 82–94% of the women treated with thermal ablation were negative for CIN2-3 after treatment; however, the duration of post-treatment follow-up was shorter relative to studies on cryotherapy. In LMICs, the efficacy of cryotherapy and thermal ablation was 83 and 82%, respectively (124).

The WHO has recommended thermal ablation for women who have histologically confirmed CIN2-3 or have screened positive in screen-and-treat strategies because of its advantages for implementation in LMICs (128). The traditional gas-based cryotherapy is difficult to implement because refrigerant gas, such as CO_2_, is expensive and difficult to procure and transport (128). Thermal ablation devices can be battery-operated, are lightweight (2–5 kg), and have a shorter treatment time (128). The use of thermal ablation in low-resource settings has been shown to be effective and acceptable (130). In Zambia, thermal ablation (44%), cryotherapy (48%), and LEEP (47%) achieved similar hrHPV clearance 6 months after treatment (131). It should be noted that up to 54% of the participants in each study arm were seropositive for HIV, and women living with HIV have a higher risk for treatment failure of precancerous lesions (131). The prevalence of treatment failure is unknown in this study, as no histological studies were performed prior to treatment. There are several ongoing and completed clinical trials on development and evaluation of the use of thermal ablation devices for LMIC settings (see NCT02956239, NCT03429582, NCT03510273 on ClinicalTrials.gov) (72).

LEEP is recommended for treating CIN2-3 if there is a medical contraindication for ablative therapy (121) or if the lesion extends into the endocervical canal (132). LEEP is often preferred over ablative techniques in high-resource settings because of its benefit of histopathologic diagnosis (132). Two meta-analyses found that 92–95% of the women treated with LEEP and 99% of the women treated with cold knife conization were free of CIN2-3 or HSIL 12 months post-treatment (Table 5) (123, 124). CIN2-3 persistence (RR: 0.87, 95% CI: 0.76–0.99) and recurrence (RR: 0.91, 95% CI: 0.84–0.99) were lower with LEEP than with cryotherapy (123).

Treatment complication rates differ by the technique. The most common adverse events associated with treatment are bleeding and infection at the cervix, which occur in <5% of treated women (126, 127, 133–135). Relative to LEEP and cold knife conization, cryotherapy and thermal ablation are cheaper, safer, and simpler to use, which makes scale-up and task-shifting more feasible, particularly in LMICs (123, 124, 136). For example, it is easier to train nurses or lay health workers to perform cryotherapy than LEEP or cold knife conization, because of the lower risk of serious complications (129). Women who previously underwent cold knife conization had the highest risk of subsequent perinatal mortality (RR: 2.87, 95% CI: 1.42–5.81) and preterm delivery at <32–34 weeks (RR: 2.78, 95% CI: 1.72–4.51), compared to the women who did not receive this procedure (136). Thermal ablation was not included in the comparisons of complications.

#### Priority Research Questions for Secondary Prevention Strategies

##### Screening and Triage of Precancerous Lesions

Point-of-care cervical cancer screening tests that facilitate same-day treatment would minimize loss to follow-up and improve continuity of care. While careHPV meets some of the REASSURED criteria (real-time connectivity, ease of specimen collection, affordable, sensitive, specific, user-friendly, rapid, equipment-free, delivered) (87, 137), and has a point-of-care platform (138), it requires batch testing (106), which enables same-day treatment only under special circumstances, such as health campaigns, and the sensitivity is significantly lower with self-sampling (109). In addition, the final cost after implementation has not been consistently affordable. For example, the per-test cost estimate was reported as $42 USD in a pilot careHPV-based screening program in Myanmar, despite some economic analyses based in low-income countries estimating costs as low as $5 USD (139). Although PCR-based point-of-care tests are currently available [e.g., GeneXpert (Cepheid, Sunnyvale, CA)] and graded pricing exists for LMICs, it is still cost-prohibitive and the required infrastructure is a barrier (140). Real-time PCR-based tests, such as AmpFire (Atila BioSystems, Inc., Mountain View, CA) (141) and Q-POC (QuantuMDx, Newcastle upon Tyne, UK) are being evaluated and developed, which will have important implications for scaling up self-sampling. More innovation is needed to make more point-of-care tests and tools affordable and widely available.

Strategies based on identifying biomarkers of HPV-associated oncogenesis are being developed to improve the precision of current screening methods and prevent the physical, psychological, and financial harms of overtreatment (142). Some examples of biomarkers include, but are not limited to, p16^ink4a^ and E6/E7 oncoproteins (121, 142–144). Tests for E6/E7 oncoproteins present a promising option for triaging screen-positive women given high positive predictive value and limited laboratory equipment (145). However, more than 60% of HPV positive women were also E6/E7 mRNA positive (146), which would present a challenge in identifying women with precancerous lesions. A systematic review of three types of HPV E6/E7 mRNA tests (Aptima, Quantivirus, and PreTect Proofer) found that while the tests have diagnostic relevance to detect CIN2-3, the higher specificity of some tests is due to the limited number of HPV types it detects (147). In a 10-year prospective cohort study in China, HPV methylation and co-testing with E6 oncoprotein showed superior area under curve values compared to cytology, viral load, and VIA (148). However, it has limitations of detecting only two HPV types. Clinician- and self-sampled first-void urine are also being evaluated as sources of viable biomarkers for detecting cervical precancers (149). In a randomized controlled trial among a Pacific Island population, HPV detection in self-collected urine (using Roche Cobas 4800 system, Roche Molecular Systems, Inc.) demonstrated moderate agreement with clinician-collected cervical samples (Kappa = 0.55, 95% CI: 0.43–0.66), with agreement for detection of hrHPV among women ages 40 and older being higher (Kappa = 0.65, 95% CI: 0.46–0.85) than that of women ages 20–39 (Kappa = 0.45, 95% CI: 0.25–0.64) (150). Formal evaluation of biomarker-based triage is needed in LMICs (145).

Several optical techniques are in development, including spectroscopy and other imaging methods (151). Such techniques have the potential to reduce the number of required visits and to save time and cost, which can be helpful especially where infrastructure for laboratories is sparse (151). For example, redesigned portable colposcopes have a high agreement with standard-of-care colposcopy for pathology (see NCT00602368 as an example) (72, 152–154). A smartphone-based colposcope can enhance VIA by taking digital images of the cervix and uploading them to an online repository for remote decision support (152). Additionally, automated visual evaluation of images with machine learning, trained using >60,000 images of the cervix from a Costa Rican tumor registry has shown greater accuracy than traditional VIA or cytology in detecting HSIL (155, 156). This algorithm can increase screening capacity and minimize subjectivity (157). Devices which could be inserted by the women herself for remote visualization of the cervix are also being developed and tested (158). However, a large (*n* = 9,406) ongoing study in Nigeria noted that the squamocolumnar junction where cancers arise was not fully visible for almost 64.6% of women by age 49 using enhanced visual assessment (MobileODT, Israel) (159). Challenges to adequate visualization of the cervix have pertinent implications for both ablative treatments and visual screening or triage efforts (159).

##### Treatment of Precancerous Lesions

Identification of women with a precancerous lesion necessitates appropriate linkage to care and treatment to prevent cancer (160). While thermal ablation has been widely recommended and is being adopted in low-resource settings, further data is needed for it to become the new standard for treating patients with precancerous lesions (161).

To optimize screen-and-treat methods, portable treatment tools, such as battery-operated cryotherapy instruments are under development (see NCT03084081) (72, 162). There are also new, portable, solar-powered (128), and battery-powered thermal ablation tools, which can be adapted to low-resource settings without stable electricity (see NCT02956239, NCT03429582) (72, 128, 162).

Photodynamic therapy has shown promising results in the treatment of CIN (163, 164). This novel technology selectively accumulates photosensitizers in pathologic tissue to destroy tumor cells by inducing necrosis (163, 165). Such technology has the potential to be a tissue-preserving treatment alternative and to minimize costs (164).

The development of therapeutic vaccines against hrHPV could help women who are already infected by stopping progression, triggering regression of lesions, and preventing recurrence of disease (166). Currently, there is no therapeutic HPV vaccine approved by the US Food and Drug Administration (167). While the use of therapeutic vaccines to treat invasive cervical cancer or other HPV-related cancers is beyond the scope of this review, many of the completed and currently ongoing trials (see NCT02481414, NCT00054041, NCT01022346, NCT03870113 as examples, not a comprehensive list) use CIN and/or HSIL as the treatment target. Smalley Rumfield et al. recently conducted a review of peptide, protein, viral vector, bacterial vector, cell, DNA, and RNA-based therapeutic vaccines as well as multi-platform and combination therapies, which demonstrate diverse potential therapies that can be useful in LMIC settings, while presenting new and different challenges (167).

In summary, the priority for secondary prevention is the optimization and scale-up of single-visit screen-and-treat modalities. Self-sampling for HPV testing offers several advantages to optimize screening coverage. Subsequent molecular oncogenic evaluation has the potential to detect lesions that are most likely to progress to cancer while reducing overtreatment. Further development is needed to simplify the testing procedure and reduce costs. The feasibility of integrating cervical cancer screening into the existing healthcare system is being explored (168, 169). For example, delivery models that leverage established HIV care infrastructure, such as staff and coordination between the clinics, can screen-and-treat women for cervical cancer to maximize efficiency (170). Other research questions related to secondary prevention based on our gap analysis include:

- Is the scale-up of current point-of-care HPV tests and HPV self-sampling effective, feasible, and cost-effective, and how can delivery models adapt sustainably to incorporate them?
- What is the most efficient and safe model of task-shifting for providing cervical cancer screening, cervical biopsy and treatment of pre-invasive disease?
- What is the optimal triage algorithm and follow-up for hrHPV positive women and those without visible precancerous lesions when biopsy is unavailable?
- Are there reliable biomarkers to predict persistent infection with hrHPV?
- Would self-visualization of the cervix as a screening tool be feasible, reliable, and acceptable?
- What are the strategies for surveillance of HPV positive women with negative oncogenic biomarkers?

### Cervical Cancer Elimination Among Women Living With HIV

#### Current State of Science

Compared to women without HIV, women living with HIV have at least 2-fold higher HPV prevalence (171, 172), experience greater persistence of HPV infection (173, 174) and have more rapid progression of precancerous lesions to HPV-associated cancers (175–178). Women living with HIV are also significantly more likely to have multiple hrHPV types detected in normal cytology, HSIL, and cervical cancer cells or tissue (179, 180). A meta-analysis showed an estimated 33,999 new cases of cervical cancer occurred among women living with HIV in 2018, corresponding to 5.8% of cases (181). In this study, women living with HIV had an overall pooled relative risk of 6.07 (95% CI: 4.40–8.37) of developing cervical cancer compared to their counterparts without HIV. When markedly immune-suppressed (i.e., CD4^+^ cell count <200 cells/ul), the risk for cervical cancer is 8-fold higher in women living with HIV compared to HIV-negative women (182). The risk of cervical cancer among women living with HIV can be mitigated to some extent with sustained antiretroviral therapy (183, 184). In a population-level analysis in Botswana, women living with HIV on antiretroviral therapy had a lower prevalence of hrHPV than those not on antiretroviral therapy (RR: 0.83, 95% CI: 0.70–0.99) (184). However, in settings with low primary and secondary prevention coverage, cervical cancer incidence and mortality among women living with HIV are high even when antiretroviral therapy is available (185). This is thought to be due to prolonged survival of effectively treated women (184).

HPV vaccines elicit high seroconversion rates and type-specific antibody levels among adolescent girls and young women living with HIV (186–195). CD4^+^ cell count at vaccination is positively correlated with seroconversion and immune response (188, 191–193). Although seropositivity and antibody levels decline more rapidly among vaccinated women living with HIV compared to vaccinated women without HIV, they are significantly higher compared to unvaccinated women living with HIV and naturally infected women without HIV (187, 190–192). As the correlate of protection against HPV is unknown, the relatively lower antibody level does not necessarily mean lower vaccine efficacy. The bivalent, quadrivalent, and nonavalent vaccines are safe for women living with HIV, as all three are virus-like particle-based vaccines (187, 192, 196).

The endpoints in most vaccine trials in HIV-positive populations are HPV seroconversion rates and immunogenicity. Studies using clinical endpoints (i.e., HPV infections and cervical abnormalities) as the outcome are still needed. The duration of protection in vaccinated adolescent girls and young women living with HIV is unknown. Extending the age of vaccination to include infants and older women could accelerate cervical cancer elimination in HIV-positive populations, although the efficiency and cost-effectiveness of such strategies depend on the prevalence of HPV infection at older ages and the duration of protection afforded by the vaccines. Context-specific modeling work would be valuable to evaluate these outcomes and inform the implementation of effective vaccination programs in settings with high HIV prevalence. In addition, the vaccine efficacy and durability of a reduced dose schedule (two or one doses) among adolescents and young adults living with HIV needs to be determined. The OPTIMO Trial, which aims to see if fewer doses can be used for children/adolescents living with HIV, will begin soon (see NCT04265950) (72).

Prevention strategies, such as VMMC can both help prevent HIV and reduce cases of cervical cancer. A modeling study of HIV prevention and HPV control in Tanzania predicts that VMMC will lower cervical cancer incidence and mortality rates by 28 and 26%, respectively, by 2070 (197).

The American Society for Clinical Oncology and the WHO recommend screening sexually active women living with HIV for HPV or cervical abnormalities as soon as they are diagnosed with HIV and rescreening within 3 years if they are HPV-negative and free of cervical lesions (121). In the United States, women living with HIV below age 30 are recommended to receive Pap screening within 1 year of onset of sexual activity regardless of the mode of HIV transmission and no later than age 21 (198). One study in the United States found that the risk of cervical cancer among regularly-screened women living with HIV was similar to HIV-negative women, highlighting the importance of screening in this population (198).

HIV presents challenges to accurate screening for women. There are more false-positive rates with VIA among women living with HIV than women without HIV (199, 200), likely due to the higher rates of cervical inflammation (201, 202). While HPV testing is an effective screening method among women living with HIV, it could lead to overestimates of cervical lesion prevalence and overtreatment (203).

Treatment failure and recurrence is more common among women living with HIV than in the general population (204). A meta-analysis found that treatment failure was twice as common among women living with HIV as among HIV-negative women (OR: 2.7, 95% CI: 2.0–3.5) (204). Women living with HIV in Sweden were five times more likely to experience recurrence than HIV-negative women (hazard ratio: 5.0, 95% CI: 2.1–11.6) (205). Women living with HIV in Kenya whose high-grade lesions were treated with cryotherapy experienced a significantly higher rate of recurrence than those treated with LEEP over 24 months (206). More research is needed to determine the most appropriate treatment method for precancerous lesions in this population.

Cervical cancer prevention services can be integrated into existing health infrastructure as women already engage in health care throughout their lifetime (e.g., antenatal care, well-child visits, and family planning. Integration of cervical cancer screening with HIV care is acceptable to women living with HIV and feasible on a small scale, however, more data are needed to determine scalability and sustainability (169).

#### Priority Research Questions for Cervical Cancer Elimination Among Women Living With HIV

- What is the duration of protection in vaccinated adolescent girls and young women living with HIV?
- What is the clinical efficacy of a reduced HPV vaccine dose (e.g., two-dose or single-dose) schedule in women living with HIV?
- What are innovative ways of effectively integrating cervical cancer prevention and treatment into HIV care?
- What is the optimal treatment method of precancerous lesions among women living with HIV?
- What are the optimal screening modalities and intervals for women living with HIV on antiretroviral therapy?

## Discussion

Global cervical cancer elimination is achievable with an increase in HPV vaccine uptake and coverage, implementation of screening and treatment strategies and emerging technologies, and development of innovative delivery approaches. Our scoping review designed a roadmap that prioritizes expanding HPV vaccination and collaborating with global organizations to allocate resources needed to eliminate cervical cancer. The focus of the concerted effort must be (1) scaling up evidence-based interventions, including the application of implementation science and measurement of population-level impact, and (2) filling the gaps through research and harnessing emerging innovations that are simple, effective, and affordable for all settings (Table 6).

**Table 6:** Innovative technologies and approaches that may be appropriate for comprehensive prevention packages.

**Priorities**	**Recommended intervention**	**Rationale**
**PRIMARY PREVENTION**		
Increase access to and coverage of HPV vaccination by the sustainable implementation of HPV immunization programs	Reduce vaccination dosage	Evidence that a single-dose is as protective as a multi-dose regimen is emerging (173, 207, 208). A single-dose regimen can be as cost-effective as the two-dose regimen, if high coverage can be achieved in low-resource settings (209, 210).
**SECONDARY PREVENTION**		
Maximize early detection of precancers and micro-invasive disease without the harms of overtreatment by increasing cervical cancer screening coverage with HPV testing and treatment starting at age 30 for at least 35 years for women without HIV	HPV testing, focusing on self-sampling	HPV DNA testing has superior sensitivity compared to cytology and VIA in detecting CIN2-3 (99, 209, 211). Self-sampling can overcome individual and structural level barriers to traditional screening methods (212, 213). It has demonstrated similar accuracy as clinician-collected samples (109, 119), and is accepted across cultures and resource-settings (214).
	Triage HPV positive women with enhanced visual assessment or a low-cost test for oncogenesis markers	The triage methods used in high-resource settings, such as cytology, colposcopy, and HPV genotyping are not ideal for low-resource settings because of their need for multiple visits, equipment, and personnel (12, 118). Innovations, such as a portable colposcope, enhanced visual assessment that utilizes mHealth and artificial intelligence, and low-cost rapid biomarker tests can accurately stratify women by the risk of progression to invasive cancer and make the process more efficient (145).
	Treat eligible precancerous lesions with thermal ablation	Thermal ablation has shown comparable efficacy to cryotherapy in treating ablation-eligible CIN2-3 in a shorter amount of time (125), and is easier to implement in LMICs than cryotherapy because it does not need CO_2_ (128) and devices are battery-operated and portable.

Lessons can be learned from the global response to HIV, which built the infrastructure that allowed for the scale-up of HIV prevention and treatment services. Since the global initiative to eliminate mother-to-child transmission of HIV was announced in 2011 (215), 80% of expecting mothers with HIV received antiretroviral therapy (ART) as part of their antenatal care (compared to 17% in 2010), and transmission dropped below 5% in several high HIV burden countries in sub-Saharan Africa (216). The joint United Nations Programme on HIV/AIDS (UNAIDS) recommended an HIV prevention “package” that combines numerous types of interventions targeting HIV transmission and treatment at multiple levels to address the various interacting risk factors of HIV (215, 217). Similar strategies can be used to combine contextually appropriate cervical cancer prevention and treatment services. Community-based clinical trials may accelerate program scale-up and increase uptake of interventions.

Following the framework of the logic model, key domestic and global stakeholders should work together to prioritize funding to procure vaccines and strengthen healthcare systems. Healthcare practitioners and communities should be engaged at every step of discussion and programmatic planning in order to build capacity and ensure successful implementation (218). Global advocacy and partnerships are needed to continue the ongoing support for HPV vaccine coverage and increased access to low-cost screening and treatment tools. For both primary and secondary prevention strategies, access to and coverage of efficacious interventions over a woman's lifetime must be prioritized. The current disparities in morbidity and mortality are likely to worsen as additional innovations emerge and are more readily adopted in high-resource than low-resource settings.

While reviewing tertiary prevention strategies (e.g., treatment of invasive cervical cancer) is beyond the scope of this article, their importance in low-resource settings cannot be overlooked. Screening implies the capacity and ethical responsibility for health agencies to make the treatment of cervical cancer available. The regions with the highest prevalence of cervical cancer have the lowest availability of skilled personnel and treatment facilities for diagnosis, surgery, chemotherapy and radiation (219, 220). The shortage of radiotherapy equipment and gynecological oncologists is a barrier to care for women with invasive cervical cancer in LMICs (220). Twenty-nine countries in Africa do not have a radiation unit (221). Gynecological oncologists are often limited to tertiary care hospitals, and women with invasive cancer have to travel considerable distances or wait for a long duration to access treatment (222). Twenty-five countries in Africa and two countries in Asia had an extreme shortage of clinical oncologists, defined as more than 1,000 incident cancers per clinical oncologist (222). Decentralization of services, where a local expert at the primary care center is supervised and mentored by a specialist, can increase access to specialty care for women living in rural or remote areas (223). Such a model can also serve as a community-based hub for dissemination of vaccines and screening efforts, such as HPV self-sampling, thereby increasing equitable access to cancer care at all levels of prevention.

The limitations of this review result from its narrative approach. Compared to systematic reviews or meta-analyses, narrative reviews are characterized by subjective study selection. In addition, due to the broad nature of the scoping review, we did not compile an exhaustive list of potentially relevant, innovative strategies and technologies. However, this paper provides an overview of the current landscape of science around cervical cancer elimination and guides the formulation of pertinent questions that deserve further exploration.

## Conclusions

The effort to eliminate cervical cancer must focus on sustainable and continuous access to prevention strategies. Large scale demonstration projects have been successfully implemented across resource settings for HPV vaccination and screen-and-treat using HPV testing and thermal ablation. Building on the lessons learned, we propose a demonstration project that combines the above-recommended strategies and provides a comprehensive cervical cancer prevention continuum to show that cervical cancer elimination can be achieved at the local level within LMICs. With a strong evidence base and effective implementation established, strategies can be scaled up more broadly. By strategically and skillfully putting scientific advances to practice, global cervical cancer elimination can be achieved.

## Author Contributions

MS and GL: methodology, data curation, writing—original draft, and writing—review and editing. NM, PG, and JW: conceptualization and writing—review and editing. DR, CB, LE, and LP: writing—review and editing. RB: conceptualization, writing—review and editing, and supervision. All authors contributed to the article and approved the submitted version.

## Conflict of Interest

The authors declare that the research was conducted in the absence of any commercial or financial relationships that could be construed as a potential conflict of interest.

## References

[1] Sung H Ferlay J Siegel RL Laversanne M Soerjomataram I Jemal A. Global cancer statistics 2020: GLOBOCAN estimates of incidence and mortality worldwide for 36 cancers in 185 countries. CA Cancer J Clin. (2021) 71:209–49. 10.3322/caac.21660 33538338

[2] World Health Organization. Cervical Cancer 2018. Available online at: https://www.who.int/cancer/prevention/diagnosis-screening/cervical-cancer/en/

[3] Peto J Gilham C Fletcher O Matthews FE. The cervical cancer epidemic that screening has prevented in the UK. Lancet. (2004) 364:249–56. 10.1016/S0140-6736(04)16674-9 15262102

[4] Canfell K Sitas F Beral V. Cervical cancer in Australia and the United Kingdom: comparison of screening policy and uptake, and cancer incidence and mortality. Med J Aust. (2006) 185:482–6. 10.5694/j.1326-5377.2006.tb00661.x 17137451

[5] Patel C Brotherton JM Pillsbury A Jayasinghe S Donovan B Macartney K. The impact of 10 years of human papillomavirus (HPV) vaccination in Australia: what additional disease burden will a nonavalent vaccine prevent? Euro Surveill. (2018) 23:1700737. 10.2807/1560-7917.ES.2018.23.41.1700737 30326995 PMC6194907

[6] World Health Organization. Global Strategy Towards the Elimination of Cervical Cancer as a Public Health Problem. World Health Organization (2020).

[7] Hall MT Simms KT Lew JB Smith MA Brotherton JM Saville M. The projected timeframe until cervical cancer elimination in Australia: a modelling study. Lancet Public Health. (2019) 4:e19–27. 10.1016/S2468-2667(18)30183-X 30291040

[8] Brisson M Kim JJ Canfell K Drolet M Gingras G Burger EA. Impact of HPV vaccination and cervical screening on cervical cancer elimination: a comparative modelling analysis in 78 low-income and lower-middle-income countries. Lancet. (2020) 395:575–90. 10.1016/S0140-6736(20)30068-4 32007141 PMC7043009

[9] Simms KT Steinberg J Caruana M Smith MA Lew JB Soerjomataram I. Impact of scaled up human papillomavirus vaccination and cervical screening and the potential for global elimination of cervical cancer in 181 countries, 2020–99: a modelling study. Lancet Oncol. (2019) 20:394–407. 10.1016/S1470-2045(18)30836-2 30795950

[10] Vaccarella S Laversanne M Ferlay J Bray F. Cervical cancer in Africa, Latin America and the Caribbean and Asia: Regional inequalities and changing trends. Int J Cancer. (2017) 141:1997–2001. 10.1002/ijc.30901 28734013

[11] Bruni L Diaz M Barrionuevo-Rosas L Herrero R Bray F Bosch FX. Global estimates of human papillomavirus vaccination coverage by region and income level: a pooled analysis. Lancet Glob Health. (2016) 4:e453–63. 10.1016/S2214-109X(16)30099-7 27340003

[12] Basu P Mittal S Bhadra Vale D Chami Kharaji Y. Secondary prevention of cervical cancer. Best Pract Res Clin Obstetr Gynaecol. (2018) 47:73–85. 10.1016/j.bpobgyn.2017.08.012 28988647

[13] Canfell K Kim JJ Brisson M Keane A Simms KT Caruana M. Mortality impact of achieving WHO cervical cancer elimination targets: a comparative modelling analysis in 78 low-income and lower-middle-income countries. Lancet. (2020) 395:591–603. 10.1016/S0140-6736(20)30157-4 32007142 PMC7043006

[14] Arksey H O'Malley L. Scoping studies: towards a methodological framework. International J Soc Res Methodol. (2005) 8:19–32. 10.1080/1364557032000119616

[15] Tricco AC Lillie E Zarin W O'Brien KK Colquhoun H Levac D. PRISMA extension for scoping reviews (PRISMA-ScR): checklist and explanation. Ann Intern Med. (2018) 169:467–73. 10.7326/M18-0850 30178033

[16] World Health Organization. Human Papillomavirus (HPV) and Cervical Cancer 2019. (2019). Available online at: https://www.who.int/news-room/fact-sheets/detail/human-papillomavirus-(hpv)-and-cervical-cancer

[17] Centers for Disease Control and Prevention. CDC Evaluation Documents, Workbooks and Tools. (2018). Available online at: https://www.cdc.gov/eval/tools/logic_models/index.html

[18] Markowitz LE Tsu V Deeks SL Cubie H Wang SA Vicari AS. Human papillomavirus vaccine introduction–the first five years. Vaccine. (2012) 30:F139–48. 10.1016/j.vaccine.2012.05.039 23199957

[19] Brusselaers N Shrestha S van de Wijgert J Verstraelen H. Vaginal dysbiosis and the risk of human papillomavirus and cervical cancer: systematic review and meta-analysis. Am J Obstetr Gynecol. (2019) 221:9–18.e8. 10.1016/j.ajog.2018.12.011 30550767

[20] Mitra A MacIntyre DA Marchesi JR Lee YS Bennett PR Kyrgiou M. The vaginal microbiota, human papillomavirus infection and cervical intraepithelial neoplasia: what do we know and where are we going next? Microbiome. (2016) 4:58. 10.1186/s40168-016-0203-0 27802830 PMC5088670

[21] Audirac-Chalifour A Torres-Poveda K Bahena-Román M Téllez-Sosa J Martínez-Barnetche J Cortina-Ceballos B. Cervical microbiome and cytokine profile at various stages of cervical cancer: a pilot study. PLoS ONE. (2016) 11:e0153274. 10.1371/journal.pone.0153274 27115350 PMC4846060

[22] Ilhan ZE Łaniewski P Thomas N Roe DJ Chase DM Herbst-Kralovetz MM. Deciphering the complex interplay between microbiota, HPV, inflammation and cancer through cervicovaginal metabolic profiling. EBioMedicine. (2019) 44:675–90. 10.1016/j.ebiom.2019.04.028 31027917 PMC6604110

[23] Paavonen J Naud P Salmeron J Wheeler CM Chow SN Apter D. Efficacy of human papillomavirus (HPV)-16/18 AS04-adjuvanted vaccine against cervical infection and precancer caused by oncogenic HPV types (PATRICIA): final analysis of a double-blind, randomised study in young women. Lancet. (2009) 374:301–14. 10.1016/S0140-6736(09)61248-4 19586656

[24] Apter D Wheeler CM Paavonen J Castellsague X Garland SM Skinner SR. Efficacy of human papillomavirus 16 and 18 (HPV-16/18) AS04-adjuvanted vaccine against cervical infection and precancer in young women: final event-driven analysis of the randomized, double-blind PATRICIA trial. Clin Vacc Immunol. (2015) 22:361–73. 10.1128/CVI.00591-14 25651922 PMC4375348

[25] Garland SM Hernandez-Avila M Wheeler CM Perez G Harper DM Leodolter S. Quadrivalent vaccine against human papillomavirus to prevent anogenital diseases. N Engl J Med. (2007) 356:1928–43. 10.1056/NEJMoa061760 17494926

[26] FUTURE II Study Group. Quadrivalent vaccine against human papillomavirus to prevent high-grade cervical lesions. N Engl J Med. (2007) 356:1915–27. 10.1056/NEJMoa061741 17494925

[27] Huh WK Joura EA Giuliano AR Iversen OE de Andrade RP Ault KA. Final efficacy, immunogenicity, and safety analyses of a nine-valent human papillomavirus vaccine in women aged 16–26 years: a randomised, double-blind trial. Lancet. (2017) 390:2143–59. 10.1016/S0140-6736(17)31821-4 28886907

[28] Joura EA Giuliano AR Iversen OE Bouchard C Mao C Mehlsen J. A 9-valent HPV vaccine against infection and intraepithelial neoplasia in women. N Engl J Med. (2015) 372:711–23. 10.1056/NEJMoa1405044 25693011

[29] Castle PE Maza M. Prophylactic HPV vaccination: past, present, and future. Epidemiol Infect. (2016) 144:449–68. 10.1017/S0950268815002198 26429676

[30] World Health Organization. Human papillomavirus vaccines: WHO position paper, October 2014—recommendations. Vaccine. (2015) 33:4383–4. 10.1016/j.vaccine.2014.12.002 25510390

[31] Brisson M Laprise JF editors. Cost-Effectiveness of Extending HPV Vaccination Above Age 26 Years in the U.S. Advisory Committee on Immunization Practices, Atlanta, GA (2019).

[32] World Health Organization. 20 Million Children Miss Out on Lifesaving Measles, Diphtheria and Tetanus Vaccines in 2018 New York. Geneva (2019). Available online at: https://www.who.int/news/item/15–07-2019–20-million-children-miss-out-on-lifesaving-measles-diphtheria-and-tetanus-vaccines-in-2018

[33] World Health Organization. Global Market Study—HPV. Geneva: World Health Organization (2018).

[34] Gavi: The Vaccine Alliance. HPV Vaccine Manufacturers Commit to Provide Enough Supply to Immunise At Least 84 Million Girls in Gavi Countries. Geneva (2020). Available online at: https://www.gavi.org/news/media-room/hpv-vaccine-manufacturers-commit-provide-enough-supply-immunise-least-84-million

[35] World Health Organization. Immunization, Vaccines and Biologicals: Data, Statistics and Graphics. Geneva (2020). Available online at: https://www.who.int/teams/immunization-vaccines-and-biologicals/data-statistics-and-graphics

[36] Kumar S Khanduri A Sidibe A Morgan C Torode J Basu P. Acting on the call: A framework for action for rapid acceleration of access to the HPV vaccination in low- and lower-middle-income countries. Int J Gynaecol Obstet. (2020) 152:32–9. 10.1002/ijgo.13482 33185283 PMC7898307

[37] GAVI: The Vaccine Alliance. Millions of Girls in Developing Countries to Be Protected Against Cervical Cancer Thanks to New HPV Vaccine Deals. (2013). Available online at: https://www.gavi.org/hpv-price-announcement

[38] Ginsberg GM Edejer TTT Lauer JA Sepulveda C. Screening, prevention and treatment of cervical cancer—a global and regional generalized cost-effectiveness analysis. Vaccine. (2009) 27:6060–79. 10.1016/j.vaccine.2009.07.026 19647813

[39] Jit M Brisson M Portnoy A Hutubessy R. Cost-effectiveness of female human papillomavirus vaccination in 179 countries: a PRIME modelling study. Lancet Glob Health. (2014) 2:e406–14. 10.1016/S2214-109X(14)70237-2 25103394

[40] Fesenfeld M Hutubessy R Jit M. Cost-effectiveness of human papillomavirus vaccination in low and middle income countries: a systematic review. Vaccine. (2013) 31:3786–804. 10.1016/j.vaccine.2013.06.060 23830973

[41] Grabowski MK Kong X Gray RH Serwadda D Kigozi G Gravitt PE. Partner human papillomavirus viral load and incident human papillomavirus detection in heterosexual couples. J Infect Dis. (2016) 213:948–56. 10.1093/infdis/jiv541 26597261 PMC4760424

[42] Wilson LE Gravitt P Tobian AA Kigozi G Serwadda D Nalugoda F. Male circumcision reduces penile high-risk human papillomavirus viral load in a randomised clinical trial in Rakai, Uganda. Sex Transm Infect. (2013) 89:262–6. 10.1136/sextrans-2012-050633 23112341 PMC3706631

[43] Wawer MJ Tobian AA Kigozi G Kong X Gravitt PE Serwadda D. Effect of circumcision of HIV-negative men on transmission of human papillomavirus to HIV-negative women: a randomised trial in Rakai, Uganda. Lancet. (2011) 377:209–18. 10.1016/S0140-6736(10)61967-8 21216000 PMC3119044

[44] Davis MA Gray RH Grabowski MK Serwadda D Kigozi G Gravitt PE. Male circumcision decreases high-risk human papillomavirus viral load in female partners: a randomized trial in Rakai, Uganda. Int J Cancer. (2013) 133:1247–52. 10.1002/ijc.28100 23400966 PMC3732529

[45] Winer RL Hughes JP Feng Q O'Reilly S Kiviat NB Holmes KK. Condom use and the risk of genital human papillomavirus infection in young women. N Engl J Med. (2006) 354:2645–54. 10.1056/NEJMoa053284 16790697

[46] Sarian LO Hammes LS Longatto-Filho A Guarisi R Derchain SF Roteli-Martins C. Increased risk of oncogenic human papillomavirus infections and incident high-grade cervical intraepithelial neoplasia among smokers: experience from the Latin American screening study. Sex Transm Dis. (2009) 36:241–8. 10.1097/OLQ.0b013e3181935a7d 19265732

[47] Eldridge RC Pawlita M Wilson L Castle PE Waterboer T Gravitt PE. Smoking and subsequent human papillomavirus infection: a mediation analysis. Ann Epidemiol. (2017) 27:724–30.e1. 10.1016/j.annepidem.2017.10.004 29107447 PMC5705255

[48] Chao XP Sun TT Wang S Fan QB Shi HH Zhu L. Correlation between the diversity of vaginal microbiota and the risk of high-risk human papillomavirus infection. Int J Gynecol Cancer. (2019) 29:28–34. 10.1136/ijgc-2018-000032 30640680

[49] Klein C Gonzalez D Samwel K Kahesa C Mwaiselage J Aluthge N. Relationship between the cervical microbiome, HIV status, and precancerous lesions. mBio. (2019) 10:e02785-18. 10.1128/mBio.02785-18 30782659 PMC6381280

[50] Kwasniewski W Wolun-Cholewa M Kotarski J Warchol W Kuzma D Kwasniewska A. Microbiota dysbiosis is associated with HPV-induced cervical carcinogenesis. Oncol Lett. (2018) 16:7035–47. 10.3892/ol.2018.9509 30546437 PMC6256731

[51] Kyrgiou M Mitra A Moscicki AB. Does the vaginal microbiota play a role in the development of cervical cancer? Transl Res. (2017) 179:168–82. 10.1016/j.trsl.2016.07.004 27477083 PMC5164950

[52] Wang H Ma Y Li R Chen X Wan L Zhao W. Associations of cervicovaginal lactobacilli with high-risk human papillomavirus infection, cervical intraepithelial neoplasia, and cancer: a systematic review and meta-analysis. J Infect Dis. (2019) 220:1243–54. 10.1093/infdis/jiz325 31242505

[53] Łaniewski P Ilhan ZE Herbst-Kralovetz MM. The microbiome and gynaecological cancer development, prevention and therapy. Nat Rev Urol. (2020) 17:232–50. 10.1038/s41585-020-0286-z 32071434 PMC9977514

[54] Attia AC Wolf J Núñez AE. On surmounting the barriers to HPV vaccination: we can do better. Ann Med. (2018) 50:209–25. 10.1080/07853890.2018.1426875 29316825

[55] Hanson CM Eckert L Bloem P Cernuschi T. Gavi HPV programs: application to implementation. Vaccines. (2015) 3:408–19. 10.3390/vaccines3020408 26343194 PMC4494350

[56] Brotherton JM Murray SL Hall MA Andrewartha LK Banks CA Meijer D. Human papillomavirus vaccine coverage among female Australian adolescents: success of the school-based approach. Med J Aust. (2013) 199:614–7. 10.5694/mja13.10272 24182228

[57] Unesco Institute for Statistics. Leaving No One Behind: How Far on the Way to Universal Primary and Secondary Education? Contract No.: Fact Sheet 37. Unesco Institute for Statistics (2016).

[58] Gallagher KE Howard N Kabakama S Mounier-Jack S Griffiths UK Feletto M. Lessons learnt from human papillomavirus (HPV) vaccination in 45 low- and middle-income countries. PLoS ONE. (2017) 12:e0177773. 10.1371/journal.pone.0177773 28575074 PMC5456063

[59] Grant LA Dunne EF Chesson H Markowitz LE. Considerations for human papillomavirus (HPV) vaccination of mid-adult women in the United States. Vaccine. (2011) 29:2365–70. 10.1016/j.vaccine.2011.01.032 21277406

[60] Tsu VD Cernuschi T LaMontagne DS. Lessons learned from HPV vaccine delivery in low-resource settings and opportunities for HIV prevention, treatment, and care among adolescents. J Acquir Immune Defic Syndr (1999). (2014) 66:S209–16. 10.1097/QAI.0000000000000175 24918597

[61] Rahman MS Gultekin M Lassi ZS. Effective approaches towards eliminating cervical cancer from low-and middle-income countries amid COVID-19 pandemic. Int J Gynecol Cancer. (2020) 30:1848. 10.1136/ijgc-2020-002013 32928927

[62] Arbyn M Bruni L Kelly D Basu P Poljak M Gultekin M. Tackling cervical cancer in Europe amidst the COVID-19 pandemic. Lancet Public Health. (2020) 5:e425. 10.1016/S2468-2667(20)30122-5 32673570 PMC7357980

[63] Elam-Evans LD Yankey D Singleton JA Sterrett N Markowitz LE Williams CL. National, regional, state, and selected local area vaccination coverage among adolescents aged 13–17 years—United States, 2019. MMWR Morb Mortal Wkly Rep. (2020) 69:1109–16. 10.15585/mmwr.mm6933a1 32817598 PMC7439984

[64] Turner TB Huh WK. HPV vaccines: Translating immunogenicity into efficacy. Hum Vacc Immunother. (2016) 12:1403–5. 10.1080/21645515.2015.1103936 26512762 PMC4964637

[65] Williamson A. S12.1 human papillomavirus vaccines—correlates of protection are not defined. Sex Transm Infect. (2013) 89:A18. 10.1136/sextrans-2013-051184.0057

[66] Secor AM Driver M Kharono B Hergott D Liu G Barnabas RV. Immunogenicity of alternative dosing schedules for HPV vaccines among adolescent girls and young women: a systematic review and meta-analysis. Vaccines. (2020) 8:618. 10.3390/vaccines8040618 33092049 PMC7712330

[67] Brotherton JM. Rationalizing the HPV vaccination schedule: a long road to a worthwhile destination. Papillomavirus Res. (2019) 8:100190. 10.1016/j.pvr.2019.100190 31759174 PMC6889720

[68] Bonner K Banura C Basta NE. HPV vaccination strategies targeting hard-to-reach populations: out-of-school girls in LMICs. Vaccine. (2018) 36:191–3. 10.1016/j.vaccine.2017.11.038 29198915

[69] Gallagher KE LaMontagne DS Watson-Jones D. Status of HPV vaccine introduction and barriers to country uptake. Vaccine. (2018) 36:4761–7. 10.1016/j.vaccine.2018.02.003 29580641

[70] Kreimer AR Struyf F Del Rosario-Raymundo MR Hildesheim A Skinner SR Wacholder S. Efficacy of fewer than three doses of an HPV-16/18 AS04-adjuvanted vaccine: combined analysis of data from the Costa Rica Vaccine and PATRICIA Trials. Lancet Oncol. (2015) 16:775–86. 10.1016/S1470-2045(15)00047-9 26071347 PMC4498478

[71] Kreimer AR Herrero R Sampson JN Porras C Lowy DR Schiller JT. Evidence for single-dose protection by the bivalent HPV vaccine–review of the Costa Rica HPV vaccine trial and future research studies. Vaccine. (2018) 36:4774–82. 10.1016/j.vaccine.2017.12.078 29366703 PMC6054558

[72] NIH, U,.S. National Library of Medicine. ClinicalTrials.gov. Available online at: https://clinicaltrials.gov/

[73] Larson HJ Jarrett C Eckersberger E Smith DMD Paterson P. Understanding vaccine hesitancy around vaccines and vaccination from a global perspective: a systematic review of published literature, 2007–2012. Vaccine. (2014) 32:2150–9. 10.1016/j.vaccine.2014.01.081 24598724

[74] Strategic Advisory Group of Experts on Immunization. Report on the Sage Working Group on Vaccine Hesitancy. Strategic Advisory Group of Experts on Immunization (2014).

[75] Gallup. Wellcome Global Monitor—First Wave Findings (2019). Available online at: https://wellcome.org/sites/default/files/wellcome-global-monitor-2018.pdf

[76] Bhopal S Nielsen M. Vaccine hesitancy in low- and middle-income countries: potential implications for the COVID-19 response. Archiv Dis Child. (2021) 106:113. 10.1136/archdischild-2020-318988 32912868

[77] Lane S MacDonald NE Marti M Dumolard L. Vaccine hesitancy around the globe: analysis of three years of WHO/UNICEF joint reporting form data-2015–2017. Vaccine. (2018) 36:3861–7. 10.1016/j.vaccine.2018.03.063 29605516 PMC5999354

[78] Vorsters A Bosch FX Bonanni P Franco EL Baay M Simas C. Prevention and control of HPV infection and HPV-related cancers in Colombia–a meeting report. BMC Proc. (2020) 14:8. 10.1186/s12919-020-00192-2 32577128 PMC7307134

[79] Castro CMM Muñoz N. HPV Vaccination in Colombia. From a Nightmare to a Bright and Promising Dawn. (2020). Available online at: www.HPVWorld.com

[80] Simas C Munoz N Arregoces L Larson HJ. HPV vaccine confidence and cases of mass psychogenic illness following immunization in Carmen de Bolivar, Colombia. Hum Vacc Immunother. (2019) 15:163–6. 10.1080/21645515.2018.1511667 30118381 PMC6363158

[81] Hanley SJB Yoshioka E Ito Y Kishi R. HPV vaccination crisis in Japan. Lancet. (2015) 385:2571. 10.1016/S0140-6736(15)61152-7 26122153

[82] Lemp JM De Neve J-W Bussmann H Chen S Manne-Goehler J Theilmann M. Lifetime prevalence of cervical cancer screening in 55 low- and middle-income countries. JAMA. (2020) 324:1532–42. 10.1001/jama.2020.16244 33079153 PMC7576410

[83] Thun M Thun M Linet MS Linet MS Cerhan JR Cerhan JR. Cancer Epidemiology and Prevention. New York, NY: Oxford University Press (2017). 10.1093/oso/9780190238667.001.0001

[84] World Health Organization. Comprehensive Cervical Cancer Control: A Guide to Essential Practice. Contract No.: Report. World Health Organization (2014). 25642554

[85] Sankaranarayanan R Nene BM Shastri SS Jayant K Muwonge R Budukh AM. HPV screening for cervical cancer in rural India. N Engl J Med. (2009) 360:1385–94. 10.1056/NEJMoa0808516 19339719

[86] Koliopoulos G Nyaga VN Santesso N Bryant A Martin-Hirsch PP Mustafa RA. Cytology versus HPV testing for cervical cancer screening in the general population. Cochrane Database Syst Rev. (2017) 8:Cd008587. 10.1002/14651858.CD008587.pub2 28796882 PMC6483676

[87] Kelly H Mayaud P Segondy M Pant Pai N Peeling RW. A systematic review and meta-analysis of studies evaluating the performance of point-of-care tests for human papillomavirus screening. Sex Transm Infect. (2017) 93:S36–45. 10.1136/sextrans-2016-053070 29223961

[88] Fokom-Domgue J Combescure C Fokom-Defo V Tebeu PM Vassilakos P Kengne AP. Performance of alternative strategies for primary cervical cancer screening in sub-Saharan Africa: systematic review and meta-analysis of diagnostic test accuracy studies. BMJ. (2015) 351:h3084. 10.1136/bmj.h3084 26142020 PMC4490835

[89] Mustafa RA Santesso N Khatib R Mustafa AA Wiercioch W Kehar R. Systematic reviews and meta-analyses of the accuracy of HPV tests, visual inspection with acetic acid, cytology, and colposcopy. Int J Gynaecol Obstetr. (2016) 132:259–65. 10.1016/j.ijgo.2015.07.024 26851054

[90] Arbyn M Sankaranarayanan R Muwonge R Keita N Dolo A Mbalawa CG. Pooled analysis of the accuracy of five cervical cancer screening tests assessed in eleven studies in Africa and India. Int J Cancer. (2008) 123:153–60. 10.1002/ijc.23489 18404671

[91] Catarino R Schafer S Vassilakos P Petignat P Arbyn M. Accuracy of combinations of visual inspection using acetic acid or lugol iodine to detect cervical precancer: a meta-analysis. BJOG. (2018) 125:545–53. 10.1111/1471-0528.14783 28603909

[92] Sankaranarayanan R Nessa A Esmy PO Dangou JM. Visual inspection methods for cervical cancer prevention. Best Pract Res Clin Obstetr Gynaecol. (2012) 26:221–32. 10.1016/j.bpobgyn.2011.08.003 22075441

[93] Fokom-Domgue J Vassilakos P Petignat P. Is screen-and-treat approach suited for screening and management of precancerous cervical lesions in Sub-Saharan Africa? Prev Med. (2014) 65:138–40. 10.1016/j.ypmed.2014.05.014 24879892

[94] Snijders PJ Verhoef VM Arbyn M Ogilvie G Minozzi S Banzi R. High-risk HPV testing on self-sampled versus clinician-collected specimens: a review on the clinical accuracy and impact on population attendance in cervical cancer screening. Int J cancer. (2013) 132:2223–36. 10.1002/ijc.27790 22907569

[95] Sauvaget C Fayette JM Muwonge R Wesley R Sankaranarayanan R. Accuracy of visual inspection with acetic acid for cervical cancer screening. Int J Gynaecol Obstetr. (2011) 113:14–24. 10.1016/j.ijgo.2010.10.012 21257169

[96] de Sanjose S Holme F. What is needed now for successful scale-up of screening? Papillomavirus Res. (2019) 7:173–5. 10.1016/j.pvr.2019.04.011 31002883 PMC6477512

[97] Jeronimo J Bansil P Lim J Peck R Paul P Amador JJ. A multicountry evaluation of careHPV testing, visual inspection with acetic acid, and papanicolaou testing for the detection of cervical cancer. Int J Gynecol Cancer. (2014) 24:576–85. 10.1097/IGC.0000000000000084 24557438 PMC4047307

[98] Ogilvie GS van Niekerk D Krajden M Smith LW Cook D Gondara L. Effect of screening with primary cervical HPV testing vs cytology testing on high-grade cervical intraepithelial neoplasia at 48 months: the HPV FOCAL randomized clinical trial. JAMA. (2018) 320:43–52. 10.1001/jama.2018.7464 29971397 PMC6583046

[99] Ronco G Giorgi Rossi P. Role of HPV DNA testing in modern gynaecological practice. Best Pract Res Clin Obstetr Gynaecol. (2018) 47:107–18. 10.1016/j.bpobgyn.2017.08.002 28918099

[100] Goodman A. HPV testing as a screen for cervical cancer. BMJ. (2015) 350:h2372. 10.1136/bmj.h2372 26126623

[101] Ronco G Dillner J Elfström KM Tunesi S Snijders PJF Arbyn M. Efficacy of HPV-based screening for prevention of invasive cervical cancer: follow-up of four European randomised controlled trials. Lancet. (2014) 383:524–32. 10.1016/S0140-6736(13)62218-7 24192252

[102] Campos NG Maza M Alfaro K Gage JC Castle PE Felix JC. The cost-effectiveness of implementing HPV testing for cervical cancer screening in El Salvador. Int J Gynaecol Obstetr. (2019) 145:40–6. 10.1002/ijgo.12773 30702142 PMC6988124

[103] Campos NG Mvundura M Jeronimo J Holme F Vodicka E Kim JJ. Cost-effectiveness of HPV-based cervical cancer screening in the public health system in Nicaragua. BMJ Open. (2017) 7:e015048. 10.1136/bmjopen-2016-015048 28619772 PMC5623348

[104] Termrungruanglert W Khemapech N Tantitamit T Sangrajrang S Havanond P Laowahutanont P. Cost-effectiveness analysis study of HPV testing as a primary cervical cancer screening in Thailand. Gynecol Oncol Rep. (2017) 22:58–63. 10.1016/j.gore.2017.09.007 29034308 PMC5633754

[105] Holme F Jeronimo J Maldonado F Camel C Sandoval M Martinez-Granera B. Introduction of HPV testing for cervical cancer screening in Central America: the Scale-Up project. Prev Med. (2020) 135:106076. 10.1016/j.ypmed.2020.106076 32247010 PMC7218710

[106] Alfaro K Maza M Felix JC Gage JC Castle PE Alonzo TA. Outcomes for step-wise implementation of a human papillomavirus testing-based cervical screen-and-treat program in El Salvador. JCO Glob Oncol. (2020) 6:1519–30. 10.1200/GO.20.00206 33064628 PMC7605377

[107] Almonte M Murillo R Sánchez GI González P Ferrera A Picconi MA. Multicentric study of cervical cancer screening with human papillomavirus testing and assessment of triage methods in Latin America: the ESTAMPA screening study protocol. BMJ Open. (2020) 10:e035796. 10.1136/bmjopen-2019-035796 32448795 PMC7252979

[108] World Health Organization. WHO Consolidated Guideline on Self-Care Interventions for Health: Sexual and Reproductive Health and Rights. Geneva: World Health Organization (2019). Available online at: https://apps.who.int/iris/bitstream/handle/10665/325480/9789241550550-eng.pdf?ua=1 31334932

[109] Arbyn M Smith SB Temin S Sultana F Castle P. Detecting cervical precancer and reaching underscreened women by using HPV testing on self samples: updated meta-analyses. BMJ. (2018) 363:k4823. 10.1136/bmj.k4823 30518635 PMC6278587

[110] Smith MA Hall MT Saville M Brotherton JM Simms KT Lew JB. Could HPV testing on self-collected samples be routinely used in an organised cervical screening program? A modelled analysis. Cancer Epidemiol Biomarkers Prev. (2020) 30:268–77. 10.1158/1055-9965.EPI-20-0998 33219163

[111] Zhang L Xu XQ Hu SY Chen F Zhang X Pan QJ. Durability of clinical performance afforded by self-collected HPV testing: a 15-year cohort study in China. Gynecol Oncol. (2018) 151:221–8. 10.1016/j.ygyno.2018.09.012 30269870

[112] Gupta S Palmer C Bik EM Cardenas JP Nuñez H Kraal L. Self-sampling for human papillomavirus testing: increased cervical cancer screening participation and incorporation in international screening programs. Front Public Health. (2018) 6:77. 10.3389/fpubh.2018.00077 29686981 PMC5900042

[113] Pimple S Mishra G Shastri S. Global strategies for cervical cancer prevention. Curr Opin Obstetr Gynecol. (2016) 28:4–10. 10.1097/GCO.0000000000000241 26642063

[114] Chatzistamatiou K Vrekoussis T Tsertanidou A Moysiadis T Mouchtaropoulou E Pasentsis K. Acceptability of self-sampling for human papillomavirus-based cervical cancer screening. J Womens Health. (2020) 29:1447–56. 10.1089/jwh.2019.8258 32757997

[115] Wong EL Cheung AW Wong AY Chan PK. Acceptability and feasibility of HPV self-sampling as an alternative primary cervical cancer screening in under-screened population groups: a cross-sectional study. Int J Environ Res Public Health. (2020) 17:6245. 10.3390/ijerph17176245 32867315 PMC7503998

[116] Odesanmi TY Wasti SP Odesanmi OS Adegbola O Oguntuase OO Mahmood S. Comparative effectiveness and acceptability of home-based and clinic-based sampling methods for sexually transmissible infections screening in females aged 14–50 years: a systematic review and meta-analysis. Sex Health. (2013) 10:559–69. 10.1071/SH13029 24160747

[117] Nelson EJ Maynard BR Loux T Fatla J Gordon R Arnold LD. The acceptability of self-sampled screening for HPV DNA: a systematic review and meta-analysis. Sex Transm Infect. (2017) 93:56–61. 10.1136/sextrans-2016-052609 28100761

[118] Mezei AK Armstrong HL Pedersen HN Campos NG Mitchell SM Sekikubo M. Cost-effectiveness of cervical cancer screening methods in low- and middle-income countries: a systematic review. Int J Cancer. (2017) 141:437–46. 10.1002/ijc.30695 28297074

[119] Yeh PT Kennedy CE de Vuyst H Narasimhan M. Self-sampling for human papillomavirus (HPV) testing: a systematic review and meta-analysis. BMJ Glob Health. (2019) 4:e001351. 10.1136/bmjgh-2018-001351 31179035 PMC6529022

[120] Arrossi S Paolino M Thouyaret L Laudi R Campanera A. Evaluation of scaling-up of HPV self-collection offered by community health workers at home visits to increase screening among socially vulnerable under-screened women in Jujuy Province, Argentina. Implement Sci. (2017) 12:17. 10.1186/s13012-017-0548-1 28193227 PMC5307871

[121] Jeronimo J Castle PE Temin S Shastri SS. Secondary prevention of cervical cancer: American Society of Clinical Oncology resource-stratified clinical practice guideline summary. J Oncol Pract. (2017) 13:129–33. 10.1200/JOP.2016.017889 27845871

[122] Kyrgiou M Mitra A Arbyn M Stasinou SM Martin-Hirsch P Bennett P. Fertility and early pregnancy outcomes after treatment for cervical intraepithelial neoplasia: systematic review and meta-analysis. BMJ. (2014) 349:g6192. 10.1136/bmj.g6192 25352501 PMC4212006

[123] D'Alessandro P Arduino B Borgo M Saccone G Venturella R Di Cello A. Loop electrosurgical excision procedure versus cryotherapy in the treatment of cervical intraepithelial neoplasia: a systematic review and meta-analysis of randomized controlled trials. Gynecol Minim Invasive Ther. (2018) 7:145–51. 10.4103/GMIT.GMIT_56_18 30306032 PMC6172872

[124] Santesso N Mustafa RA Wiercioch W Kehar R Gandhi S Chen Y. Systematic reviews and meta-analyses of benefits and harms of cryotherapy, LEEP, and cold knife conization to treat cervical intraepithelial neoplasia. Int J Gynaecol Obstetr. (2016) 132:266–71. 10.1016/j.ijgo.2015.07.026 26643302

[125] de Fouw M Oosting RM Rutgrink A Dekkers OM Peters AAW Beltman JJ. A systematic review and meta-analysis of thermal coagulation compared with cryotherapy to treat precancerous cervical lesions in low- and middle-income countries. Int J Gynaecol Obstetr. (2019) 147:4–18. 10.1002/ijgo.12904 31273785

[126] Sauvaget C Muwonge R Sankaranarayanan R. Meta-analysis of the effectiveness of cryotherapy in the treatment of cervical intraepithelial neoplasia. Int J Gynaecol Obstetr. (2013) 120:218–23. 10.1016/j.ijgo.2012.10.014 23265830

[127] Randall TC Sauvaget C Muwonge R Trimble EL Jeronimo J. Worthy of further consideration: an updated meta-analysis to address the feasibility, acceptability, safety and efficacy of thermal ablation in the treatment of cervical cancer precursor lesions. Prev Med. (2019) 118:81–91. 10.1016/j.ypmed.2018.10.006 30342109

[128] World Health Organization. WHO Guidelines for the Use of Thermal Ablation for Cervical Pre-cancer Lesions. (2019). Available online at: https://apps.who.int/iris/bitstream/handle/10665/329299/9789241550598-eng.pdf?sequence=1&isAllowed=y 31661202

[129] Basu P Taghavi K Hu S-Y Mogri S Joshi S. Management of cervical premalignant lesions. Curr Probl Cancer. (2018) 42:129–36. 10.1016/j.currproblcancer.2018.01.010 29428790

[130] Papoutsis D Underwood M Parry-Smith W Panikkar J. Early and late pregnancy outcomes in women treated with cold-coagulation versus LLETZ cervical treatment for cervical intraepithelial neoplasia; a retrospective cohort study. Archiv Gynecol Obstetr. (2018) 297:1015–25. 10.1007/s00404-018-4704-x 29404740

[131] Pinder LF Parham GP Basu P Muwonge R Lucas E Nyambe N. Thermal ablation versus cryotherapy or loop excision to treat women positive for cervical precancer on visual inspection with acetic acid test: pilot phase of a randomised controlled trial. Lancet Oncol. (2020) 21:175–84. 10.1016/S1470-2045(19)30635-7 31734069 PMC6946855

[132] World Health Organization. WHO Guidelines Approved by the Guidelines Review Committee. WHO Guidelines for Treatment of Cervical Intraepithelial Neoplasia 2–3 and Adenocarcinoma in situ: Cryotherapy, Large Loop Excision of the Transformation Zone, and Cold Knife Conization. Geneva: World Health Organization (2014). 24901204

[133] Naud PS Muwonge R Passos EP Magno V Matos J Sankaranarayanan R. Efficacy, safety, and acceptability of thermocoagulation for treatment of cervical intraepithelial neoplasia in a hospital setting in Brazil. Int J Gynaecol Obstetr. (2016) 133:351–4. 10.1016/j.ijgo.2015.09.035 27005927

[134] Viviano M Kenfack B Catarino R Tincho E Temogne L Benski AC. Feasibility of thermocoagulation in a screen-and-treat approach for the treatment of cervical precancerous lesions in sub-Saharan Africa. BMC Womens Health. (2017) 17:2. 10.1186/s12905-016-0355-x 28061842 PMC5219781

[135] Dolman L Sauvaget C Muwonge R Sankaranarayanan R. Meta-analysis of the efficacy of cold coagulation as a treatment method for cervical intraepithelial neoplasia: a systematic review. BJOG. (2014) 121:929–42. 10.1111/1471-0528.12655 24597779

[136] Arbyn M Kyrgiou M Simoens C Raifu AO Koliopoulos G Martin-Hirsch P. Perinatal mortality and other severe adverse pregnancy outcomes associated with treatment of cervical intraepithelial neoplasia: meta-analysis. BMJ. (2008) 337:a1284. 10.1136/bmj.a1284 18801868 PMC2544379

[137] Land KJ Boeras DI Chen XS Ramsay AR Peeling RW. Reassured diagnostics to inform disease control strategies, strengthen health systems and improve patient outcomes. Nat Microbiol. (2019) 4:46–54. 10.1038/s41564-018-0295-3 30546093 PMC7097043

[138] Sayed S Chung M Temmerman M. Point-of-care HPV molecular diagnostics for a test-and-treat model in high-risk HIV populations. Lancet Glob Health. (2020) 8:e171–2. 10.1016/S2214-109X(19)30559-5 31981548

[139] Tin-Oo C Hlaing HN Nandar CS Aung T Fishbein D. Why the cost of purchasing the careHPV test in Myanmar was many times greater than that reported in the international literature. J Glob Oncol. (2018) 4:49s. 10.1200/jgo.18.27900

[140] Cubie HA Campbell C. Cervical cancer screening—the challenges of complete pathways of care in low-income countries: focus on Malawi. Womens Health. (2020) 16:1745506520914804. 10.1177/1745506520914804 32364058 PMC7225784

[141] Zhang W Du H Huang X Wang C Duan X Liu Y. Evaluation of an isothermal amplification HPV detection assay for primary cervical cancer screening. Infect Agent Cancer. (2020) 15:65. 10.1186/s13027-020-00328-1 33110442 PMC7583687

[142] Sahasrabuddhe VV Luhn P Wentzensen N. Human papillomavirus and cervical cancer: biomarkers for improved prevention efforts. Future Microbiol. (2011) 6:1083–98. 10.2217/fmb.11.87 21958146 PMC3809085

[143] Wentzensen N Clarke MA Bremer R Poitras N Tokugawa D Goldhoff PE. Clinical evaluation of human papillomavirus screening with p16/Ki-67 dual stain triage in a large organized cervical cancer screening program. JAMA Intern Med. (2019) 179:881–8. 10.1001/jamainternmed.2019.0306 31081870 PMC6515572

[144] Nuovo GJ de Andrade CV Wells SI Brusadelli M Nicol AF. New biomarkers of human papillomavirus infection in acute cervical intraepithelial neoplasia. Ann Diagn Pathol. (2018) 36:21–7. 10.1016/j.anndiagpath.2018.06.008 29966832

[145] Wentzensen N Schiffman M Palmer T Arbyn M. Triage of HPV positive women in cervical cancer screening. J Clin Virol. (2016) 76:S49–55. 10.1016/j.jcv.2015.11.015 26643050 PMC4789103

[146] Rossi PG Carozzi F Ronco G Allia E Bisanzi S Gillio-Tos A. p16/ki67 and E6/E7 mRNA accuracy and prognostic value in triaging HPV DNA-positive women. J Natl Cancer Inst. (2020) 113:292–300. 10.1093/jnci/djaa105 32745170 PMC7936054

[147] Derbie A Mekonnen D Woldeamanuel Y Van Ostade X Abebe T. HPV E6/E7 mRNA test for the detection of high grade cervical intraepithelial neoplasia (CIN2+): a systematic review. Infect Agents Cancer. (2020) 15:9. 10.1186/s13027-020-0278-x 32047531 PMC7006188

[148] Dong L Zhang L Hu S-Y Feng R-M Zhao X-L Zhang Q. Risk stratification of HPV 16 DNA methylation combined with E6 oncoprotein in cervical cancer screening: a 10-year prospective cohort study. Clin Epigenet. (2020) 12:62. 10.1186/s13148-020-00853-1 32381054 PMC7204324

[149] Arbyn M Peeters E Benoy I Vanden Broeck D Bogers J De Sutter P. VALHUDES: a protocol for validation of human papillomavirus assays and collection devices for HPV testing on self-samples and urine samples. J Clin Virol. (2018) 107:52–6. 10.1016/j.jcv.2018.08.006 30195193

[150] Hernandez BY Tareg AC Reichhardt M Agapito A Zhu X Sy A. Randomized controlled trial evaluating the utility of urine HPV DNA for cervical cancer screening in a Pacific Island population. J Glob Health Rep. (2018) 2:e2018016. 10.29392/joghr.2.e2018016 30542667 PMC6287926

[151] Novikova T. Optical techniques for cervical neoplasia detection. Beilstein J Nanotechnol. (2017) 8:1844–62. 10.3762/bjnano.8.186 29046833 PMC5629403

[152] Lam CT Mueller J Asma B Asiedu M Krieger MS Chitalia R. An integrated strategy for improving contrast, durability, and portability of a pocket colposcope for cervical cancer screening and diagnosis. PLoS ONE. (2018) 13:e0192530. 10.1371/journal.pone.0192530 29425225 PMC5806883

[153] Mueller JL Lam CT Dahl D Asiedu MN Krieger MS Bellido-Fuentes Y. Portable Pocket colposcopy performs comparably to standard-of-care clinical colposcopy using acetic acid and Lugol's iodine as contrast mediators: an investigational study in Peru. BJOG. (2018) 125:1321–9. 10.1111/1471-0528.15326 29893472 PMC6115285

[154] Mueller JL Asma E Lam CT Krieger MS Gallagher JE Erkanli A. International image concordance study to compare a point-of-Care Tampon colposcope with a standard-of-care colposcope. J Lower Genit Tract Dis. (2017) 21:112–9. 10.1097/LGT.0000000000000306 28263237 PMC5365351

[155] Peterson CW Rose D Mink J Levitz D. Real-time monitoring and evaluation of a visual-based cervical cancer screening program using a decision support job aid. Diagnostics (Basel). (2016) 6:20. 10.3390/diagnostics6020020 27196932 PMC4931415

[156] Hu L Bell D Antani S Xue Z Yu K Horning MP. An observational study of deep learning and automated evaluation of cervical images for cancer screening. J Natl Cancer Inst. (2019) 111:923–32. 10.1093/jnci/djy225 30629194 PMC6748814

[157] Asiedu MN Agudogo J Krieger MS Miros R Proeschold-Bell RJ Schmitt JW. Design and preliminary analysis of a vaginal inserter for speculum-free cervical cancer screening. PLoS ONE. (2017) 12:e0177782. 10.1371/journal.pone.0177782 28562669 PMC5451045

[158] Asiedu MN Agudogo JS Dotson ME Krieger MS Schmitt JW Huchko M. A novel, versatile speculum-free callascope for clinical examination and self-visualization of the cervix. bioRxiv. (2019) 2019:618348. 10.1101/618348

[159] Desai KT Ajenifuja KO Banjo A Adepiti CA Novetsky A Sebag C. Design and feasibility of a novel program of cervical screening in Nigeria: self-sampled HPV testing paired with visual triage. Infect Agent Cancer. (2020) 15:60. 10.1186/s13027-020-00324-5 33072178 PMC7556552

[160] LaVigne AW Triedman SA Randall TC Trimble EL Viswanathan AN. Cervical cancer in low and middle income countries: addressing barriers to radiotherapy delivery. Gynecol Oncol Rep. (2017) 22:16–20. 10.1016/j.gore.2017.08.004 28948205 PMC5602511

[161] Petignat P Kenfack B. Is thermal ablation a new standard for cervical pre-cancer treatment in low-income and middle-income countries? Lancet Oncol. (2020) 21:19–20. 10.1016/S1470-2045(19)30732-6 31734070

[162] Castle PE Murokora D Perez C Alvarez M Quek SC Campbell C. Treatment of cervical intraepithelial lesions. Int J Gynaecol Obstetr. (2017) 138:20–5. 10.1002/ijgo.12191 28691333

[163] Hillemanns P Garcia F Petry KU Dvorak V Sadovsky O Iversen OE. A randomized study of hexaminolevulinate photodynamic therapy in patients with cervical intraepithelial neoplasia 1/2. Am J Obstetr Gynecol. (2015) 212:465.e1–7. 10.1016/j.ajog.2014.10.1107 25467012

[164] Istomin YP Lapzevich TP Chalau VN Shliakhtsin SV Trukhachova TV. Photodynamic therapy of cervical intraepithelial neoplasia grades II and III with photolon. Photodiagn Photodyn Ther. (2010) 7:144–51. 10.1016/j.pdpdt.2010.06.005 20728837

[165] Soergel P Löning M Staboulidou I Schippert C Hillemanns P. Photodynamic diagnosis and therapy in gynecology. J Environ Pathol Toxicol Oncol. (2008) 27:307–20. 10.1615/JEnvironPatholToxicolOncol.v27.i4.80 19105537

[166] Kisling K Zhang L Simonds H Fakie N Yang J McCarroll R. Fully automatic treatment planning for external-beam radiation therapy of locally advanced cervical cancer: a tool for low-resource clinics. J Glob Oncol. (2019) 5:1–9. 10.1200/JGO.18.00107 30629457 PMC6426517

[167] Smalley Rumfield C Roller N Pellom ST Schlom J Jochems C. Therapeutic vaccines for HPV-associated malignancies. Immunotargets Ther. (2020) 9:167–200. 10.2147/ITT.S273327 33117742 PMC7549137

[168] Rubin R. Artificial intelligence for cervical precancer screening. JAMA. (2019) 321:734. 10.1001/jama.2019.0888 30806677

[169] Sigfrid L Murphy G Haldane V Chuah FLH Ong SE Cervero-Liceras F. Integrating cervical cancer with HIV healthcare services: a systematic review. PLoS ONE. (2017) 12:e0181156. 10.1371/journal.pone.0181156 28732037 PMC5521786

[170] Davies N Chersich M Mullick S Naidoo N Makhoba N Rees H. Integrating cervical cancer screening into safer conception services to improve women's health outcomes: a pilot study at a primary care clinic in South Africa. Sex Transm Dis. (2019) 46:91–7. 10.1097/OLQ.0000000000000914 30308532 PMC6336485

[171] Morhard R Nief C Barrero Castedo C Hu F Madonna M Mueller JL. Development of enhanced ethanol ablation as an alternative to surgery in treatment of superficial solid tumors. Sci Rep. (2017) 7:8750. 10.1038/s41598-017-09371-2 28821832 PMC5562881

[172] Poljak M Sterbenc A Lunar MM. Prevention of human papillomavirus (HPV)-related tumors in people living with human immunodeficiency virus (HIV). Expert Rev Antiinfect Ther. (2017) 15:987–99. 10.1080/14787210.2017.1392854 29027811

[173] Safaeian M Sampson JN Pan Y Porras C Kemp TJ Herrero R. Durability of protection afforded by fewer doses of the HPV16/18 vaccine: the CVT trial. J Natl Cancer Inst. (2017) 110:205–12. 10.1093/jnci/djx158 28954299 PMC6075614

[174] Phelan DF Gange SJ Ahdieh-Grant L Mehta SH Kirk GD Shah K. Determinants of newly detected human papillomavirus infection in HIV-infected and HIV-uninfected injection drug using women. Sex Transm Dis. (2009) 36:149–56. 10.1097/OLQ.0b013e31818d3df3 19174735 PMC2888629

[175] Strickler HD Burk RD Fazzari M Anastos K Minkoff H Massad LS. Natural history and possible reactivation of human papillomavirus in human immunodeficiency virus-positive women. J Natl Cancer Inst. (2005) 97:577–86. 10.1093/jnci/dji073 15840880

[176] Kuhn L Wang C Tsai WY Wright TC Denny L. Efficacy of human papillomavirus-based screen-and-treat for cervical cancer prevention among HIV-infected women. AIDS. (2010) 24:2553–61. 10.1097/QAD.0b013e32833e163e 20706107

[177] Ezechi OC Ostergren PO Nwaokorie FO Ujah IA Odberg Pettersson K. The burden, distribution and risk factors for cervical oncogenic human papilloma virus infection in HIV positive Nigerian women. Virol J. (2014) 11:5. 10.1186/1743-422X-11-5 24433568 PMC3896716

[178] Cholli P Bradford L Manga S Nulah K Kiyang E Manjuh F. Screening for cervical cancer among HIV-positive and HIV-negative women in Cameroon using simultaneous co-testing with careHPV DNA testing and visual inspection enhanced by digital cervicography: findings of initial screening and one-year follow-up. Gynecol Oncol. (2018) 148:118–25. 10.1016/j.ygyno.2017.11.002 29153541

[179] Moscicki AB Ellenberg JH Farhat S Xu J. Persistence of human papillomavirus infection in HIV-infected and -uninfected adolescent girls: risk factors and differences, by phylogenetic type. J Infect Dis. (2004) 190:37–45. 10.1086/421467 15195241

[180] Clifford GM de Vuyst H Tenet V Plummer M Tully S Franceschi S. Effect of HIV infection on human papillomavirus types causing invasive cervical cancer in Africa. J Acquir Immune Defic Syndr (1999). (2016) 73:332–9. 10.1097/QAI.0000000000001113 27331659 PMC5172520

[181] Stelzle D Tanaka LF Lee KK Ibrahim Khalil A Baussano I Shah ASV. Estimates of the global burden of cervical cancer associated with HIV. Lancet Glob Health. (2020) 9:e161–9. 10.1016/S2214-109X(20)30459-9 33212031 PMC7815633

[182] Menon S Wusiman A Boily MC Kariisa M Mabeya H Luchters S. Epidemiology of HPV genotypes among HIV positive women in Kenya: a systematic review and meta-analysis. PLoS ONE. (2016) 11:e0163965. 10.1371/journal.pone.0163965 27764092 PMC5072621

[183] Goedert JJ Hosgood HD Biggar RJ Strickler HD Rabkin CS. Screening for cancer in persons living with HIV infection. Trends Cancer. (2016) 2:416–28. 10.1016/j.trecan.2016.06.007 27891533 PMC5120729

[184] Kelly H Weiss HA Benavente Y de Sanjose S Mayaud P. Association of antiretroviral therapy with high-risk human papillomavirus, cervical intraepithelial neoplasia, and invasive cervical cancer in women living with HIV: a systematic review and meta-analysis. Lancet HIV. (2018) 5:e45–58. 10.1016/S2352-3018(17)30149-2 29107561 PMC5757426

[185] Dryden-Peterson S Bvochora-Nsingo M Suneja G Efstathiou JA Grover S Chiyapo S. HIV infection and survival among women with cervical cancer. J Clin Oncol. (2016) 34:3749–57. 10.1200/JCO.2016.67.9613 27573661 PMC5477924

[186] Dryden-Peterson S Medhin H Kebabonye-Pusoentsi M Seage GR III Suneja G Kayembe MK. Cancer incidence following expansion of HIV treatment in Botswana. PLoS ONE. (2015) 10:e0135602. 10.1371/journal.pone.0135602 26267867 PMC4534370

[187] Kahn JA Xu J Kapogiannis BG Rudy B Gonin R Liu N. Immunogenicity and safety of the human papillomavirus 6, 11, 16, 18 vaccine in HIV-infected young women. Clin Infect Dis. (2013) 57:735–44. 10.1093/cid/cit319 23667266 PMC3739463

[188] Kojic EM Kang M Cespedes MS Umbleja T Godfrey C Allen RT. Immunogenicity and safety of the quadrivalent human papillomavirus vaccine in HIV-1-infected women. Clin Infect Dis. (2014) 59:127–35. 10.1093/cid/ciu238 24723284 PMC4305143

[189] Giacomet V Penagini F Trabattoni D Vigano A Rainone V Bernazzani G. Safety and immunogenicity of a quadrivalent human papillomavirus vaccine in HIV-infected and HIV-negative adolescents and young adults. Vaccine. (2014) 32:5657–61. 10.1016/j.vaccine.2014.08.011 25149430

[190] McClymont E Lee M Raboud J Coutlee F Walmsley S Lipsky N. The efficacy of the quadrivalent human papillomavirus vaccine in girls and women living with human immunodeficiency virus. Clin Infect Dis. (2019) 68:788–94. 10.1093/cid/ciy575 29985988

[191] Mugo NR Eckert L Magaret AS Cheng A Mwaniki L Ngure K. Quadrivalent HPV vaccine in HIV-1-infected early adolescent girls and boys in Kenya: month 7 and 12 post vaccine immunogenicity and correlation with immune status. Vaccine. (2018) 36:7025–32. 10.1016/j.vaccine.2018.09.059 30297124

[192] Levin MJ Moscicki AB Song LY Fenton T Meyer WA III Read JS. Safety and immunogenicity of a quadrivalent human papillomavirus (types 6, 11, 16, and 18) vaccine in HIV-infected children 7 to 12 years old. J Acquir Immune Defic Syndr (1999). (2010) 55:197–204. 10.1097/QAI.0b013e3181de8d26 20574412 PMC3033215

[193] Weinberg A Song LY Saah A Brown M Moscicki AB Meyer WA III. Humoral, mucosal, and cell-mediated immunity against vaccine and nonvaccine genotypes after administration of quadrivalent human papillomavirus vaccine to HIV-infected children. J Infect Dis. (2012) 206:1309–18. 10.1093/infdis/jis489 22859825 PMC3529604

[194] Toft L Storgaard M Muller M Sehr P Bonde J Tolstrup M. Comparison of the immunogenicity and reactogenicity of Cervarix and Gardasil human papillomavirus vaccines in HIV-infected adults: a randomized, double-blind clinical trial. J Infect Dis. (2014) 209:1165–73. 10.1093/infdis/jit657 24273179

[195] Faust H Toft L Sehr P Muller M Bonde J Forslund O. Human papillomavirus neutralizing and cross-reactive antibodies induced in HIV-positive subjects after vaccination with quadrivalent and bivalent HPV vaccines. Vaccine. (2016) 34:1559–65. 10.1016/j.vaccine.2016.02.019 26896686

[196] Denny L Hendricks B Gordon C Thomas F Hezareh M Dobbelaere K. Safety and immunogenicity of the HPV-16/18 AS04-adjuvanted vaccine in HIV-positive women in South Africa: a partially-blind randomised placebo-controlled study. Vaccine. (2013) 31:5745–53. 10.1016/j.vaccine.2013.09.032 24091311

[197] Hall MT Smith MA Simms KT Barnabas RV Canfell K Murray JM. The past, present and future impact of HIV prevention and control on HPV and cervical disease in Tanzania: a modelling study. PLoS ONE. (2020) 15:e0231388. 10.1371/journal.pone.0231388 32374729 PMC7202618

[198] HIV PoOIiAaAw. Guidelines for the Prevention and Treatment of Opportunistic Infections in Adults and Adolescents With HIV: Recommendations From the Centers for Disease Control and Prevention, the National Institutes of Health, and the HIV Medicine Association of the Infectious Diseases Society of America. AIDSinfo (2018). Available online at: http://aidsinfo.nih.gov/contentfiles/lvguidelines/adult_oi.pdf

[199] Sahasrabuddhe VV Bhosale RA Kavatkar AN Nagwanshi CA Joshi SN Jenkins CA. Comparison of visual inspection with acetic acid and cervical cytology to detect high-grade cervical neoplasia among HIV-infected women in India. Int J Cancer. (2012) 130:234–40. 10.1002/ijc.25971 21387289 PMC3516675

[200] Mutyaba T Mirembe F Sandin S Weiderpass E. Evaluation of ‘see-see and treat' strategy and role of HIV on cervical cancer prevention in Uganda. Reprod Health. (2010) 7:4. 10.1186/1742-4755-7-4 20459733 PMC2882355

[201] Sahasrabuddhe VV Parham GP Mwanahamuntu MH Vermund SH. Cervical Cancer prevention in low- and middle-income countries: feasible, affordable, essential. Cancer Prev Res. (2012) 5:11–7. 10.1158/1940-6207.CAPR-11-0540 22158053 PMC3586242

[202] Menon S Rossi R Harmon SG Mabeya H Callens S. Public health approach to prevent cervical cancer in HIV-infected women in Kenya: issues to consider in the design of prevention programs. Gynecol Oncol Rep. (2017) 22:82–8. 10.1016/j.gore.2017.10.002 29159260 PMC5678735

[203] Mapanga W Girdler-Brown B Feresu SA Chipato T Singh E. Prevention of cervical cancer in HIV-seropositive women from developing countries through cervical cancer screening: a systematic review. Syst Rev. (2018) 7:198. 10.1186/s13643-018-0874-7 30447695 PMC6240280

[204] Debeaudrap P Sobngwi J Tebeu P-M Clifford GM. Residual or recurrent precancerous lesions after treatment of cervical lesions in human immunodeficiency virus-infected women: a systematic review and meta-analysis of treatment failure. Clin Infect Dis. (2019) 69:1555–65. 10.1093/cid/ciy1123 30602038 PMC6792085

[205] Carlander C Wagner P van Beirs A Yilmaz A Elfgren K Dillner J. Suppressive antiretroviral therapy associates with effective treatment of high-grade cervical intraepithelial neoplasia. AIDS. (2018) 32:1475–84. 10.1097/QAD.0000000000001853 29746299

[206] Greene SA De Vuyst H John-Stewart GC Richardson BA McGrath CJ Marson KG. Effect of cryotherapy vs loop electrosurgical excision procedure on cervical disease recurrence among women with HIV and high-grade cervical lesions in Kenya: a randomized clinical trial. JAMA. (2019) 322:1570–9. 10.1001/jama.2019.14969 31638680 PMC6806442

[207] Chido-Amajuoyi OG Domgue JF Obi-Jeff C Schmeler K Shete S. A call for the introduction of gender-neutral HPV vaccination to national immunisation programmes in Africa. Lancet Glob Health. (2019) 7:e20–1. 10.1016/S2214-109X(18)30405-4 30514615

[208] Sankaranarayanan R Prabhu PR Pawlita M Gheit T Bhatla N Muwonge R. Immunogenicity and HPV infection after one, two, and three doses of quadrivalent HPV vaccine in girls in India: a multicentre prospective cohort study. Lancet Oncol. (2016) 17:67–77. 10.1016/S1470-2045(15)00414-3 26652797 PMC5357737

[209] Burger EA Campos NG Sy S Regan C Kim JJ. Health and economic benefits of single-dose HPV vaccination in a Gavi-eligible country. Vaccine. (2018) 36:4823–9. 10.1016/j.vaccine.2018.04.061 29807710 PMC6066173

[210] Whitworth HS Gallagher KE Howard N Mounier-Jack S Mbwanji G Kreimer AR. Efficacy and immunogenicity of a single dose of human papillomavirus vaccine compared to no vaccination or standard three and two-dose vaccination regimens: a systematic review of evidence from clinical trials. Vaccine. (2020) 38:1302–14. 10.1016/j.vaccine.2019.12.017 31870572

[211] Umulisa MC Franceschi S Baussano I Tenet V Uwimbabazi M Rugwizangoga B. Evaluation of human-papillomavirus testing and visual inspection for cervical cancer screening in Rwanda. BMC Womens Health. (2018) 18:59. 10.1186/s12905-018-0549-5 29699549 PMC5921370

[212] Majid U Kandasamy S Farrah K Vanstone M. Women's preferences and experiences of cervical cancer screening in rural and remote areas: a systematic review and qualitative meta-synthesis. Rural Remote Health. (2019) 19:5190. 10.22605/RRH5190 31640391

[213] Ndejjo R Mukama T Kiguli J Musoke D. Knowledge, facilitators and barriers to cervical cancer screening among women in Uganda: a qualitative study. BMJ Open. (2017) 7:e016282. 10.1136/bmjopen-2017-016282 28606908 PMC5541520

[214] Madzima TR Vahabi M Lofters A. Emerging role of HPV self-sampling in cervical cancer screening for hard-to-reach women: focused literature review. Can Fam Physician. (2017) 63:597–601. Available online at: https://www.cfp.ca/content/63/8/597/tab-article-info 28807952 PMC5555324

[215] Joint United Nations Programme on HIV/AIDS (UNAIDS). Global Plan Towards the Elimination of New HIV Infections Among Children by 2015 and Keeping Their Mothers Alive, 2011–2015. Geneva: UNAIDS (2011).

[216] United Nations Children's Fund. Women: At the Heart of the HIV Response for Children. New York, NY: UNICEF (2018).

[217] (UNAIDS) JUNPoHA. Combination HIV Prevention: Tailoring and Coordinating Biomedical, Behavioural and Structural Strategies to Reduce New HIV Infections—A UNAIDS Discussion Paper. Geneva: UNAIDS (2010).

[218] Tapela NM Mpunga T Hedt-Gauthier B Moore M Mpanumusingo E Xu MJ. Pursuing equity in cancer care: implementation, challenges and preliminary findings of a public cancer referral center in rural Rwanda. BMC Cancer. (2016) 16:237. 10.1186/s12885-016-2256-7 26992690 PMC4797361

[219] Sullivan R Alatise OI Anderson BO Audisio R Autier P Aggarwal A. Global cancer surgery: delivering safe, affordable, and timely cancer surgery. Lancet Oncol. (2015) 16:1193–224. 10.1016/S1470-2045(15)00223-5 26427363

[220] Wu ES Jeronimo J Feldman S. Barriers and challenges to treatment alternatives for early-stage cervical cancer in lower-resource settings. J Glob Oncol. (2017) 3:572–82. 10.1200/JGO.2016.007369 29094097 PMC5646895

[221] Grover S Xu MJ Yeager A Rosman L Groen RS Chackungal S. A systematic review of radiotherapy capacity in low- and middle-income countries. Front Oncol. (2015) 4:380. 10.3389/fonc.2014.00380 25657930 PMC4302829

[222] Mathew A. Global survey of clinical oncology workforce. J Glob Oncol. (2018) 4:1–12. 10.1200/JGO.17.00188 30241241 PMC6223442

[223] Gago J Pendharkar D Tripathi C Ginsburg O. Making the best use of resources in global cancer care. Am Soc Clin Oncol Educ Book. (2020) 40:e361–6. 10.1200/EDBK_290311 32614655

