# Supplementary material for: A Framework for Cervical Cancer Elimination in Low-and-Middle-Income Countries: A Scoping Review and Roadmap for Interventions and Research Priorities
Source: Front Public Health. 2021 Jul 1;9:670032. doi: 10.3389/fpubh.2021.670032 (PMC8281011; doi:10.3389/fpubh.2021.670032)
Supplement: Supplementary file 1 [file Table_1.docx]

**S1 Appendix. Search results**

| Prevention node | Prevention strategy | Database | Search strategy/keywords | Total number of studies retrieved | Date of retrieval | Number of studies included in the manuscript |
| --- | --- | --- | --- | --- | --- | --- |
| Primary prevention | HPV vaccination | PubMed | ((("Uterine Cervical Neoplasms"[Mesh]) AND (("2006/1/1"[Date - Publication] : "2019/1/25"[Date - Publication])) AND ((ffrft[Filter]) AND (meta-analysis[Filter] OR randomizedcontrolledtrial[Filter] OR systematicreview[Filter])) AND ((ffrft[Filter]) AND (meta-analysis[Filter] OR randomizedcontrolledtrial[Filter] OR systematicreview[Filter]))) AND ((ffrft[Filter]) AND (booksdocs[Filter] OR clinicaltrial[Filter] OR clinicaltrialphasei[Filter] OR clinicaltrialphaseii[Filter] OR clinicaltrialphaseiii[Filter] OR clinicaltrialphaseiv[Filter] OR controlledclinicaltrial[Filter] OR journalarticle[Filter] OR meta-analysis[Filter] OR multicenterstudy[Filter] OR observationalstudy[Filter] OR pragmaticclinicaltrial[Filter] OR randomizedcontrolledtrial[Filter] OR review[Filter] OR systematicreview[Filter]) AND (fft[Filter]) AND (english[Filter])) AND ((ffrft[Filter]) AND (booksdocs[Filter] OR clinicaltrial[Filter] OR clinicaltrialphasei[Filter] OR clinicaltrialphaseii[Filter] OR clinicaltrialphaseiii[Filter] OR clinicaltrialphaseiv[Filter] OR controlledclinicaltrial[Filter] OR journalarticle[Filter] OR meta-analysis[Filter] OR multicenterstudy[Filter] OR observationalstudy[Filter] OR pragmaticclinicaltrial[Filter] OR randomizedcontrolledtrial[Filter] OR review[Filter] OR systematicreview[Filter]) AND (fft[Filter]) AND (english[Filter]))) AND (hpv vaccine[MeSH Terms]) | 107 | 21-Jan-20 | 20 |
|  |  | Embase | ('hpv'/exp OR hpv) AND vaccin* AND ('efficacy'/exp OR efficacy OR effectiveness OR 'sensitivity'/exp OR sensitivity OR 'specificity'/exp OR specificity) AND (('meta analysis'/de OR 'randomized controlled trial'/de OR 'systematic review'/de)) AND [2010-2020]/py | 408 | 5-Jan-21 |  |
|  | Tobacco cessation | PubMed | Search: (("Uterine Cervical Neoplasms"[Mesh]) AND (("2006/1/1"[Date - Publication] : "2019/1/25"[Date - Publication])) AND ((ffrft[Filter]) AND (meta-analysis[Filter] OR randomizedcontrolledtrial[Filter] OR systematicreview[Filter])) AND ((ffrft[Filter]) AND (meta-analysis[Filter] OR randomizedcontrolledtrial[Filter] OR systematicreview[Filter]))) AND (tobacco)  Filters: Free full text, Full text, Books and Documents, Clinical Trial, Clinical Trial, Phase I, Clinical Trial, Phase II, Clinical Trial, Phase III, Clinical Trial, Phase IV, Controlled Clinical Trial, Journal Article, Meta-Analysis, Multicenter Study, Observational Study, Pragmatic Clinical Trial, Randomized Controlled Trial, Review, Systematic Review, English | 8 | 21-Jan-20 | 1 |
|  |  | Embase | (cervical AND cancer) AND (tobacco AND cessation) OR (tobacco AND educat*) AND ('meta analysis'/de OR 'randomized controlled trial'/de OR 'systematic review'/de) AND ('article'/it OR 'conference abstract'/it OR 'conference paper'/it OR 'conference review'/it OR 'review'/it) AND ('efficacy'/exp OR efficacy OR effectiveness OR 'sensitivity'/exp OR sensitivity OR 'specificity'/exp OR specificity) AND [2010-2020]/py | 3 | 5-Jan-21 |  |
|  |  | Embase | (cervical AND cancer) AND ((tobacco AND cessation) OR (tobacco AND educat*)) AND ('efficacy'/exp OR efficacy OR effectiveness OR 'sensitivity'/exp OR sensitivity OR 'specificity'/exp OR specificity) AND [2010-2020]/py | 19 | 5-Jan-21 |  |
|  | Sexual education | PubMed | ((("Uterine Cervical Neoplasms"[Mesh]) AND (("2006/1/1"[Date - Publication] : "2019/1/25"[Date - Publication])) AND ((ffrft[Filter]) AND (meta-analysis[Filter] OR randomizedcontrolledtrial[Filter] OR systematicreview[Filter])) AND ((ffrft[Filter]) AND (meta-analysis[Filter] OR randomizedcontrolledtrial[Filter] OR systematicreview[Filter]))) AND ((ffrft[Filter]) AND (booksdocs[Filter] OR clinicaltrial[Filter] OR clinicaltrialphasei[Filter] OR clinicaltrialphaseii[Filter] OR clinicaltrialphaseiii[Filter] OR clinicaltrialphaseiv[Filter] OR controlledclinicaltrial[Filter] OR journalarticle[Filter] OR meta-analysis[Filter] OR multicenterstudy[Filter] OR observationalstudy[Filter] OR pragmaticclinicaltrial[Filter] OR randomizedcontrolledtrial[Filter] OR review[Filter] OR systematicreview[Filter]) AND (fft[Filter]) AND (english[Filter])) AND ((ffrft[Filter]) AND (booksdocs[Filter] OR clinicaltrial[Filter] OR clinicaltrialphasei[Filter] OR clinicaltrialphaseii[Filter] OR clinicaltrialphaseiii[Filter] OR clinicaltrialphaseiv[Filter] OR controlledclinicaltrial[Filter] OR journalarticle[Filter] OR meta-analysis[Filter] OR multicenterstudy[Filter] OR observationalstudy[Filter] OR pragmaticclinicaltrial[Filter] OR randomizedcontrolledtrial[Filter] OR review[Filter] OR systematicreview[Filter]) AND (fft[Filter]) AND (english[Filter]))) AND (sex education) | 5 | 21-Jan-20 | 0 |
|  |  | Embase | (cervical AND cancer) AND (sex* AND education) AND ('meta analysis'/de OR 'randomized controlled trial'/de OR 'systematic review'/de) AND ('article'/it OR 'conference abstract'/it OR 'conference paper'/it OR 'conference review'/it OR 'review'/it) AND ('efficacy'/exp OR efficacy OR effectiveness OR 'sensitivity'/exp OR sensitivity OR 'specificity'/exp OR specificity) AND [2010-2020]/py | 12 | 5-Jan-21 |  |
|  |  | Embase | (cervical AND cancer) AND (sex* AND education) AND ('efficacy'/exp OR efficacy OR effectiveness OR 'sensitivity'/exp OR sensitivity OR 'specificity'/exp OR specificity) AND [2010-2020]/py | 153 | 5-Jan-21 |  |
|  | Condoms | PubMed | ((("Uterine Cervical Neoplasms"[Mesh]) AND (("2006/1/1"[Date - Publication] : "2019/1/25"[Date - Publication])) AND ((ffrft[Filter]) AND (meta-analysis[Filter] OR randomizedcontrolledtrial[Filter] OR systematicreview[Filter])) AND ((ffrft[Filter]) AND (meta-analysis[Filter] OR randomizedcontrolledtrial[Filter] OR systematicreview[Filter]))) AND ((ffrft[Filter]) AND (booksdocs[Filter] OR clinicaltrial[Filter] OR clinicaltrialphasei[Filter] OR clinicaltrialphaseii[Filter] OR clinicaltrialphaseiii[Filter] OR clinicaltrialphaseiv[Filter] OR controlledclinicaltrial[Filter] OR journalarticle[Filter] OR meta-analysis[Filter] OR multicenterstudy[Filter] OR observationalstudy[Filter] OR pragmaticclinicaltrial[Filter] OR randomizedcontrolledtrial[Filter] OR review[Filter] OR systematicreview[Filter]) AND (fft[Filter]) AND (english[Filter])) AND ((ffrft[Filter]) AND (booksdocs[Filter] OR clinicaltrial[Filter] OR clinicaltrialphasei[Filter] OR clinicaltrialphaseii[Filter] OR clinicaltrialphaseiii[Filter] OR clinicaltrialphaseiv[Filter] OR controlledclinicaltrial[Filter] OR journalarticle[Filter] OR meta-analysis[Filter] OR multicenterstudy[Filter] OR observationalstudy[Filter] OR pragmaticclinicaltrial[Filter] OR randomizedcontrolledtrial[Filter] OR review[Filter] OR systematicreview[Filter]) AND (fft[Filter]) AND (english[Filter]))) AND (condom) | 5 | 21-Jan-20 | 2 |
|  |  | Embase | (cervical AND cancer) AND (condom) AND ('meta analysis'/de OR 'randomized controlled trial'/de OR 'systematic review'/de) AND ('article'/it OR 'conference abstract'/it OR 'conference paper'/it OR 'conference review'/it OR 'review'/it) AND ('efficacy'/exp OR efficacy OR effectiveness OR 'sensitivity'/exp OR sensitivity OR 'specificity'/exp OR specificity) AND [2010-2020]/py | 6 | 5-Jan-21 |  |
|  |  | Embase | (cervical AND cancer) AND (condom) AND ('efficacy'/exp OR efficacy OR effectiveness OR 'sensitivity'/exp OR sensitivity OR 'specificity'/exp OR specificity) AND [2010-2020]/py | 53 | 5-Jan-21 |  |
|  | Voluntary medical male circumcision | PubMed | ((("Uterine Cervical Neoplasms"[Mesh]) AND (("2006/1/1"[Date - Publication] : "2019/1/25"[Date - Publication])) AND ((ffrft[Filter]) AND (meta-analysis[Filter] OR randomizedcontrolledtrial[Filter] OR systematicreview[Filter])) AND ((ffrft[Filter]) AND (meta-analysis[Filter] OR randomizedcontrolledtrial[Filter] OR systematicreview[Filter]))) AND ((ffrft[Filter]) AND (booksdocs[Filter] OR clinicaltrial[Filter] OR clinicaltrialphasei[Filter] OR clinicaltrialphaseii[Filter] OR clinicaltrialphaseiii[Filter] OR clinicaltrialphaseiv[Filter] OR controlledclinicaltrial[Filter] OR journalarticle[Filter] OR meta-analysis[Filter] OR multicenterstudy[Filter] OR observationalstudy[Filter] OR pragmaticclinicaltrial[Filter] OR randomizedcontrolledtrial[Filter] OR review[Filter] OR systematicreview[Filter]) AND (fft[Filter]) AND (english[Filter])) AND ((ffrft[Filter]) AND (booksdocs[Filter] OR clinicaltrial[Filter] OR clinicaltrialphasei[Filter] OR clinicaltrialphaseii[Filter] OR clinicaltrialphaseiii[Filter] OR clinicaltrialphaseiv[Filter] OR controlledclinicaltrial[Filter] OR journalarticle[Filter] OR meta-analysis[Filter] OR multicenterstudy[Filter] OR observationalstudy[Filter] OR pragmaticclinicaltrial[Filter] OR randomizedcontrolledtrial[Filter] OR review[Filter] OR systematicreview[Filter]) AND (fft[Filter]) AND (english[Filter]))) AND (male circumcision) | 2 | 21-Jan-20 | 3 |
|  |  | Embase | (cervical AND cancer) AND (male AND circumcision) AND ('meta analysis'/de OR 'randomized controlled trial'/de OR 'systematic review'/de) AND ('article'/it OR 'conference abstract'/it OR 'conference paper'/it OR 'conference review'/it OR 'review'/it) AND ('efficacy'/exp OR efficacy OR effectiveness OR 'sensitivity'/exp OR sensitivity OR 'specificity'/exp OR specificity) AND [2010-2020]/py | 1 | 5-Jan-21 |  |
|  |  | Embase | (cervical AND cancer) AND (male AND circumcision) AND ('efficacy'/exp OR efficacy OR effectiveness OR 'sensitivity'/exp OR sensitivity OR 'specificity'/exp OR specificity) AND [2010-2020]/py | 10 | 5-Jan-21 |  |
| Secondary prevention | HPV test | PubMed | ((("Uterine Cervical Neoplasms"[Mesh]) AND (("2006/1/1"[Date - Publication] : "2019/1/25"[Date - Publication])) AND ((ffrft[Filter]) AND (meta-analysis[Filter] OR randomizedcontrolledtrial[Filter] OR systematicreview[Filter])) AND ((ffrft[Filter]) AND (meta-analysis[Filter] OR randomizedcontrolledtrial[Filter] OR systematicreview[Filter]))) AND ((ffrft[Filter]) AND (booksdocs[Filter] OR clinicaltrial[Filter] OR clinicaltrialphasei[Filter] OR clinicaltrialphaseii[Filter] OR clinicaltrialphaseiii[Filter] OR clinicaltrialphaseiv[Filter] OR controlledclinicaltrial[Filter] OR journalarticle[Filter] OR meta-analysis[Filter] OR multicenterstudy[Filter] OR observationalstudy[Filter] OR pragmaticclinicaltrial[Filter] OR randomizedcontrolledtrial[Filter] OR review[Filter] OR systematicreview[Filter]) AND (fft[Filter]) AND (english[Filter])) AND ((ffrft[Filter]) AND (booksdocs[Filter] OR clinicaltrial[Filter] OR clinicaltrialphasei[Filter] OR clinicaltrialphaseii[Filter] OR clinicaltrialphaseiii[Filter] OR clinicaltrialphaseiv[Filter] OR controlledclinicaltrial[Filter] OR journalarticle[Filter] OR meta-analysis[Filter] OR multicenterstudy[Filter] OR observationalstudy[Filter] OR pragmaticclinicaltrial[Filter] OR randomizedcontrolledtrial[Filter] OR review[Filter] OR systematicreview[Filter]) AND (fft[Filter]) AND (english[Filter]))) AND (hpv test*) | 185 | 21-Jan-20 | 18 |
|  |  | Embase | (cervical AND cancer) AND (hpv AND test*) AND ('meta analysis'/de OR 'randomized controlled trial'/de OR 'systematic review'/de) AND ('article'/it OR 'conference abstract'/it OR 'conference paper'/it OR 'conference review'/it OR 'review'/it) AND ('efficacy'/exp OR efficacy OR effectiveness OR 'sensitivity'/exp OR sensitivity OR 'specificity'/exp OR specificity) AND [2010-2020]/py | 245 | 5-Jan-21 |  |
|  | VIA | PubMed | ((("Uterine Cervical Neoplasms"[Mesh]) AND (("2006/1/1"[Date - Publication] : "2019/1/25"[Date - Publication])) AND ((ffrft[Filter]) AND (meta-analysis[Filter] OR randomizedcontrolledtrial[Filter] OR systematicreview[Filter])) AND ((ffrft[Filter]) AND (meta-analysis[Filter] OR randomizedcontrolledtrial[Filter] OR systematicreview[Filter]))) AND ((ffrft[Filter]) AND (booksdocs[Filter] OR clinicaltrial[Filter] OR clinicaltrialphasei[Filter] OR clinicaltrialphaseii[Filter] OR clinicaltrialphaseiii[Filter] OR clinicaltrialphaseiv[Filter] OR controlledclinicaltrial[Filter] OR journalarticle[Filter] OR meta-analysis[Filter] OR multicenterstudy[Filter] OR observationalstudy[Filter] OR pragmaticclinicaltrial[Filter] OR randomizedcontrolledtrial[Filter] OR review[Filter] OR systematicreview[Filter]) AND (fft[Filter]) AND (english[Filter])) AND ((ffrft[Filter]) AND (booksdocs[Filter] OR clinicaltrial[Filter] OR clinicaltrialphasei[Filter] OR clinicaltrialphaseii[Filter] OR clinicaltrialphaseiii[Filter] OR clinicaltrialphaseiv[Filter] OR controlledclinicaltrial[Filter] OR journalarticle[Filter] OR meta-analysis[Filter] OR multicenterstudy[Filter] OR observationalstudy[Filter] OR pragmaticclinicaltrial[Filter] OR randomizedcontrolledtrial[Filter] OR review[Filter] OR systematicreview[Filter]) AND (fft[Filter]) AND (english[Filter]))) AND (visual inspection with acetic acid OR VIA) | 59 | 21-Jan-20 | 10 |
|  |  | Embase | (cervical AND cancer) AND (visual AND inspection AND with AND acetic AND acid OR via) AND ('meta analysis'/de OR 'randomized controlled trial'/de OR 'systematic review'/de) AND ('article'/it OR 'conference abstract'/it OR 'conference paper'/it OR 'conference review'/it OR 'review'/it) AND ('efficacy'/exp OR efficacy OR effectiveness OR 'sensitivity'/exp OR sensitivity OR 'specificity'/exp OR specificity) AND [2010-2020]/py | 118 | 5-Jan-21 |  |
|  | Cytology | PubMed | ((("Uterine Cervical Neoplasms"[Mesh]) AND (("2006/1/1"[Date - Publication] : "2019/1/25"[Date - Publication])) AND ((ffrft[Filter]) AND (meta-analysis[Filter] OR randomizedcontrolledtrial[Filter] OR systematicreview[Filter])) AND ((ffrft[Filter]) AND (meta-analysis[Filter] OR randomizedcontrolledtrial[Filter] OR systematicreview[Filter]))) AND ((ffrft[Filter]) AND (booksdocs[Filter] OR clinicaltrial[Filter] OR clinicaltrialphasei[Filter] OR clinicaltrialphaseii[Filter] OR clinicaltrialphaseiii[Filter] OR clinicaltrialphaseiv[Filter] OR controlledclinicaltrial[Filter] OR journalarticle[Filter] OR meta-analysis[Filter] OR multicenterstudy[Filter] OR observationalstudy[Filter] OR pragmaticclinicaltrial[Filter] OR randomizedcontrolledtrial[Filter] OR review[Filter] OR systematicreview[Filter]) AND (fft[Filter]) AND (english[Filter])) AND ((ffrft[Filter]) AND (booksdocs[Filter] OR clinicaltrial[Filter] OR clinicaltrialphasei[Filter] OR clinicaltrialphaseii[Filter] OR clinicaltrialphaseiii[Filter] OR clinicaltrialphaseiv[Filter] OR controlledclinicaltrial[Filter] OR journalarticle[Filter] OR meta-analysis[Filter] OR multicenterstudy[Filter] OR observationalstudy[Filter] OR pragmaticclinicaltrial[Filter] OR randomizedcontrolledtrial[Filter] OR review[Filter] OR systematicreview[Filter]) AND (fft[Filter]) AND (english[Filter]))) AND (pap OR cytology) | 301 | 21-Jan-20 | 3 |
|  |  | Embase | (cervical AND cancer) AND (cytology OR pap) AND ('meta analysis'/de OR 'randomized controlled trial'/de OR 'systematic review'/de) AND ('article'/it OR 'conference abstract'/it OR 'conference paper'/it OR 'conference review'/it OR 'review'/it) AND ('efficacy'/exp OR efficacy OR effectiveness OR 'sensitivity'/exp OR sensitivity OR 'specificity'/exp OR specificity) AND [2010-2020]/py | 304 | 5-Jan-21 |  |
|  | Thermal ablation | PubMed | ((("Uterine Cervical Neoplasms"[Mesh]) AND (("2006/1/1"[Date - Publication] : "2019/1/25"[Date - Publication])) AND ((ffrft[Filter]) AND (meta-analysis[Filter] OR randomizedcontrolledtrial[Filter] OR systematicreview[Filter])) AND ((ffrft[Filter]) AND (meta-analysis[Filter] OR randomizedcontrolledtrial[Filter] OR systematicreview[Filter]))) AND ((ffrft[Filter]) AND (booksdocs[Filter] OR clinicaltrial[Filter] OR clinicaltrialphasei[Filter] OR clinicaltrialphaseii[Filter] OR clinicaltrialphaseiii[Filter] OR clinicaltrialphaseiv[Filter] OR controlledclinicaltrial[Filter] OR journalarticle[Filter] OR meta-analysis[Filter] OR multicenterstudy[Filter] OR observationalstudy[Filter] OR pragmaticclinicaltrial[Filter] OR randomizedcontrolledtrial[Filter] OR review[Filter] OR systematicreview[Filter]) AND (fft[Filter]) AND (english[Filter])) AND ((ffrft[Filter]) AND (booksdocs[Filter] OR clinicaltrial[Filter] OR clinicaltrialphasei[Filter] OR clinicaltrialphaseii[Filter] OR clinicaltrialphaseiii[Filter] OR clinicaltrialphaseiv[Filter] OR controlledclinicaltrial[Filter] OR journalarticle[Filter] OR meta-analysis[Filter] OR multicenterstudy[Filter] OR observationalstudy[Filter] OR pragmaticclinicaltrial[Filter] OR randomizedcontrolledtrial[Filter] OR review[Filter] OR systematicreview[Filter]) AND (fft[Filter]) AND (english[Filter]))) AND (thermal ablation OR thermocoagulation) | 2 | 21-Jan-20 | 11 |
|  |  | Embase | (cervical AND cancer) AND (thermal AND ablation OR thermocoagulation) AND ('meta analysis'/de OR 'randomized controlled trial'/de OR 'systematic review'/de) AND ('article'/it OR 'conference abstract'/it OR 'conference paper'/it OR 'conference review'/it OR 'review'/it) AND ('efficacy' OR effectiveness) AND [2010-2020]/py | 12 | 5-Jan-21 |  |
|  | Cryotherapy | PubMed | ((("Uterine Cervical Neoplasms"[Mesh]) AND (("2006/1/1"[Date - Publication] : "2019/1/25"[Date - Publication])) AND ((ffrft[Filter]) AND (meta-analysis[Filter] OR randomizedcontrolledtrial[Filter] OR systematicreview[Filter])) AND ((ffrft[Filter]) AND (meta-analysis[Filter] OR randomizedcontrolledtrial[Filter] OR systematicreview[Filter]))) AND ((ffrft[Filter]) AND (booksdocs[Filter] OR clinicaltrial[Filter] OR clinicaltrialphasei[Filter] OR clinicaltrialphaseii[Filter] OR clinicaltrialphaseiii[Filter] OR clinicaltrialphaseiv[Filter] OR controlledclinicaltrial[Filter] OR journalarticle[Filter] OR meta-analysis[Filter] OR multicenterstudy[Filter] OR observationalstudy[Filter] OR pragmaticclinicaltrial[Filter] OR randomizedcontrolledtrial[Filter] OR review[Filter] OR systematicreview[Filter]) AND (fft[Filter]) AND (english[Filter])) AND ((ffrft[Filter]) AND (booksdocs[Filter] OR clinicaltrial[Filter] OR clinicaltrialphasei[Filter] OR clinicaltrialphaseii[Filter] OR clinicaltrialphaseiii[Filter] OR clinicaltrialphaseiv[Filter] OR controlledclinicaltrial[Filter] OR journalarticle[Filter] OR meta-analysis[Filter] OR multicenterstudy[Filter] OR observationalstudy[Filter] OR pragmaticclinicaltrial[Filter] OR randomizedcontrolledtrial[Filter] OR review[Filter] OR systematicreview[Filter]) AND (fft[Filter]) AND (english[Filter]))) AND (cryotherapy) | 10 | 21-Jan-20 | 7 |
|  |  | Embase | (cervical AND cancer) AND (cryotherapy) AND ('meta analysis'/de OR 'randomized controlled trial'/de OR 'systematic review'/de) AND ('article'/it OR 'conference abstract'/it OR 'conference paper'/it OR 'conference review'/it OR 'review'/it) AND [2010-2020]/py | 67 | 5-Jan-21 |  |
|  | LEEP | PubMed | ((("Uterine Cervical Neoplasms"[Mesh]) AND (("2006/1/1"[Date - Publication] : "2019/1/25"[Date - Publication])) AND ((ffrft[Filter]) AND (meta-analysis[Filter] OR randomizedcontrolledtrial[Filter] OR systematicreview[Filter])) AND ((ffrft[Filter]) AND (meta-analysis[Filter] OR randomizedcontrolledtrial[Filter] OR systematicreview[Filter]))) AND ((ffrft[Filter]) AND (booksdocs[Filter] OR clinicaltrial[Filter] OR clinicaltrialphasei[Filter] OR clinicaltrialphaseii[Filter] OR clinicaltrialphaseiii[Filter] OR clinicaltrialphaseiv[Filter] OR controlledclinicaltrial[Filter] OR journalarticle[Filter] OR meta-analysis[Filter] OR multicenterstudy[Filter] OR observationalstudy[Filter] OR pragmaticclinicaltrial[Filter] OR randomizedcontrolledtrial[Filter] OR review[Filter] OR systematicreview[Filter]) AND (fft[Filter]) AND (english[Filter])) AND ((ffrft[Filter]) AND (booksdocs[Filter] OR clinicaltrial[Filter] OR clinicaltrialphasei[Filter] OR clinicaltrialphaseii[Filter] OR clinicaltrialphaseiii[Filter] OR clinicaltrialphaseiv[Filter] OR controlledclinicaltrial[Filter] OR journalarticle[Filter] OR meta-analysis[Filter] OR multicenterstudy[Filter] OR observationalstudy[Filter] OR pragmaticclinicaltrial[Filter] OR randomizedcontrolledtrial[Filter] OR review[Filter] OR systematicreview[Filter]) AND (fft[Filter]) AND (english[Filter]))) AND (LEEP) | 7 | 21-Jan-20 | 6 |
|  |  | Embase | (cervical AND cancer) AND (leep OR 'loop electrosurgical excision') AND ('meta analysis'/de OR 'randomized controlled trial'/de OR 'systematic review'/de) AND ('article'/it OR 'conference abstract'/it OR 'conference paper'/it OR 'conference review'/it OR 'review'/it) AND [2010-2020]/py | 37 | 5-Jan-21 |  |
|  | HPV self-sampling | PubMed | ((("Uterine Cervical Neoplasms"[Mesh]) AND (("2006/1/1"[Date - Publication] : "2019/1/25"[Date - Publication])) AND ((ffrft[Filter]) AND (meta-analysis[Filter] OR randomizedcontrolledtrial[Filter] OR systematicreview[Filter])) AND ((ffrft[Filter]) AND (meta-analysis[Filter] OR randomizedcontrolledtrial[Filter] OR systematicreview[Filter]))) AND ((ffrft[Filter]) AND (booksdocs[Filter] OR clinicaltrial[Filter] OR clinicaltrialphasei[Filter] OR clinicaltrialphaseii[Filter] OR clinicaltrialphaseiii[Filter] OR clinicaltrialphaseiv[Filter] OR controlledclinicaltrial[Filter] OR journalarticle[Filter] OR meta-analysis[Filter] OR multicenterstudy[Filter] OR observationalstudy[Filter] OR pragmaticclinicaltrial[Filter] OR randomizedcontrolledtrial[Filter] OR review[Filter] OR systematicreview[Filter]) AND (fft[Filter]) AND (english[Filter])) AND ((ffrft[Filter]) AND (booksdocs[Filter] OR clinicaltrial[Filter] OR clinicaltrialphasei[Filter] OR clinicaltrialphaseii[Filter] OR clinicaltrialphaseiii[Filter] OR clinicaltrialphaseiv[Filter] OR controlledclinicaltrial[Filter] OR journalarticle[Filter] OR meta-analysis[Filter] OR multicenterstudy[Filter] OR observationalstudy[Filter] OR pragmaticclinicaltrial[Filter] OR randomizedcontrolledtrial[Filter] OR review[Filter] OR systematicreview[Filter]) AND (fft[Filter]) AND (english[Filter]))) AND (HPV self-sampl* OR HPV self-collect*) | 50 | 21-Jan-20 | 13 |
|  |  | Embase | (hpv AND 'self sampl*' OR hpv) AND 'self collect*' AND (cervical AND cancer) AND ('meta analysis'/de OR 'randomized controlled trial'/de OR 'systematic review'/de) AND ('article'/it OR 'conference abstract'/it OR 'conference paper'/it OR 'conference review'/it OR 'review'/it) AND [2010-2020]/py | 57 | 5-Jan-21 |  |
