# Supplementary material for: A Framework for Cervical Cancer Elimination in Low-and-Middle-Income Countries: A Scoping Review and Roadmap for Interventions and Research Priorities
Source: Front Public Health. 2021 Jul 1;9:670032. doi: 10.3389/fpubh.2021.670032 (PMC8281011; doi:10.3389/fpubh.2021.670032)
Supplement: Supplementary file 2 [file Table_2.docx]

**S2 Appendix. Data abstraction**

|  | **Author** | **Year** | **Study name** | **Study Design** | **Location** | **Population** | **Intervention** | **Intervnetion # dose** | **Comparison** | **Comparison # dose** | **Outcomes** | **Sample size** |
| --- | --- | --- | --- | --- | --- | --- | --- | --- | --- | --- | --- | --- |
| Efficacy | Huh et al.(1) | 2017 | “Final efficacy, immunogenicity, and safety analyses of a nine-valent human papillomavirus vaccine in women aged 16-26 years: a randomised, double-blind trial” | RCT | 18 countries | 16-26yo women | 9vHPV | 3 (0, 2, 6) | 4vHPV | 3 (0, 2, 6) | HSIL, persistent HPV infections, antibody titer | 14,215  9vHPV (n=7,106)  4vHPV (n=7,109) |
|  | Paavonen et al.(2) | 2009 | PATRICIA | RCT | 14 countries | 15-25 yo women | 2vHPV | 3 (0, 1, 6) | Hep A vaccine | 3 (0, 1, 6) | CIN2+, persistent infections with 16/18 and other HR types | 16,162  Vaccine (n=8,093)  Control (n=8,069) |
|  | Apter et al.(3) | 2015 | PATRICIA | RCT | 14 countries | 15-25 yo women | 2vHPV | 3 (0, 1, 6) | Hep A vaccine | 3 (0, 1, 6) | CIN2+, persistent infections with 16/18 and other HR types | 18,644  Vaccine (n=9,319)  Control (n=9,325) |
|  | Garland et al.(4) | 2007 | FUTURES I | RCT | 16 countries | 16-24yo women | 4vHPV | 3 | Placebo | 3 | Any CIN or AIS Antibody titer | 5,455  Vaccine (n=2,723)  Control (n=2,732) |
|  | Futures II study group(5) | 2007 | FUTURES II | RCT | 16 countries | 15-26 yo women | 4vHPV | 3 | Placebo | 3 | HSIL or CIN2+, AIS, CC | 12,167  Vaccine (n=5,305)  Control (n=5260) |
|  | Kahn et al.(6) | 2013 | “Immunogenicity and safety of the human papillomavirus 6, 11, 16, 18 vaccine in HIV-infected young women” | RCT | US and PR | HIV+ 16-23yo women | 4vHPV | 3 (0, 2, 6) | HIV-neg women | 3 (0, 2, 6) | Antibody titer | 366  Vaccine (n=69; no ART  n=30; ART)  Control (n=267 HIV negative) |
|  | Kojic et al.(7) | 2014 | AIDS Clinical Trials Group | RCT | US, Brazil, South Africa | HIV+  13-45yo women | 4vHPV | 3 (0, 2, 6) | Stratum A (>350 Cells/µL)  Stratum B (201–350 Cells/µL)  Stratum C (≤200 Cells/µL) | 3 (0, 2, 6) | Immunogenicity | 315  Stratum A (n=127)  Stratum B (n=95)  Stratum C (n=93) |
|  | Giacomet et al.(8) | 2014 | “Safety and immunogenicity of a quadrivalent human papillomavirus vaccine in HIV-infected and HIV-negative adolescents and young adults” | Matched cohort | Italy | HIV+  13-27yo men and women | 4vHPV | 3 (0, 2, 6) | HIV-neg | 3 (0, 2, 6) | Immunogenicity | 92  Virally suppressed HIV+ (n=46)  HIV negative (n=46) |
|  | McClymont et al.(9) | 2019 | HPV in HIV Study Group | Cohort | Canada | HIV+  13-66yo women | 4vHPV | 3 (0, 2, 6) | Unvaccinated HIV+  Unvaccinated HIV- | 3 (0, 2, 6) | 6 month persistent HPV CIN2+ | 279 |
|  | Mugo et al.(10) | 2018 | “Quadrivalent HPV vaccine in HIV-1-infected early adolescent girls and boys in Kenya: Month 7 and 12 post vaccine immunogenicity and correlation with immune status” | Noninferiority | Kenya | HIV+  9-14yo girls and boys | 4vHPV | 3 (0, 2, 6) | Vaccinated historical HIV+ and HIV- controls | 3 (0, 2, 6) | Immunogenicity | 189  Girls (n=100)  Boys (n=80) |
|  | Levin et al.(11) | 2010 | IMPAACT PACTG P1047 | RCT | US | HIV+  7-12yo girls and boys | 4vHPV | 3 (0, 2, 6) | Placebo | 3 (0, 2, 6) | Immunogenicity | 126  4vHPV (n=96)  Placebo (n=30) |
|  | Weinberg et al.(12) | 2012 | IMPAACT PACTG P1047 | RCT | N/A | HIV+  7-12yo girls and boys | 4vHPV | 3 (0, 2, 6) + booster at 24 months | Delayed 4vHPV | 3 (0, 2, 6) | Immunogenicity | 126  Immediate injection (n=96)  Delayed (n=30) |
|  | Toft et al.(13) | 2014 | “Comparison of the immunogenicity of Cervarix® and Gardasil® human papillomavirus vaccines for oncogenic non-vaccine serotypes HPV-31, HPV-33, and HPV-45 in HIV-infected adults” | RCT | Denmark | HIV+  men and women, median age 44.5-47.0 | 4vHPV | 3 (0, 1.5, 6) | 2vHPV | 3 (0, 1.5, 6) | Immunogenicity | 91  4vHPV (n=46)  2vHPV (n=45) |
|  | Faust et al.(14) | 2016 | “Human Papillomavirus neutralizing and cross-reactive antibodies induced in HIV-positive subjects after vaccination with quadrivalent and bivalent HPV vaccines” | RCT | Denmark | HIV+  men and women, median age 44.5-47.0 | 4vHPV | 3 (0, 1.5, 6) | 2vHPV | 3 (0, 1.5, 6) | Seroconversion rate | 91  4vHPV (n=46)  2vHPV (n=45) |
|  | Arbyn, Xu (15) | 2018 | “Efficacy and safety of prophylactic HPV vaccines. A Cochrane review of randomized trials” | Meta-analysis | Global | N/A | Any HPV vaccine types | N/A | N/A | N/A | 1) CIN2+, CIN3+, and AIS related to the HPV types included in the vaccine  2) Any CIN2+, CIN3+, and AIS irrespective of HPV types | 26 trials |
|  | Signorelli et al.(16) | 2017 | “Human papillomavirus 9-valent vaccine for cancer prevention: a systematic review of the available evidence” | Systematic review | All studies were multi-center multi-countries trials | N/A | 9vHPV | N/A | N/A | N/A | Efficacy, immunogenicity and safety, and registered, completed, and ongoing RCTs | 10 articles |
| Population effectiveness | Patel et al.(17) | 2018 | “The impact of 10 years of human papillomavirus (HPV) vaccination in Australia: what additional disease burden will a nonavalent vaccine prevent?” | Non-systematic literature review | Australia | N/A | Any HPV vaccine types | N/A | N/A | N/A | Burden of HPV-associated cancers and diseases in Australia | N/A |
|  | Steben et al.(18) | 2018 | “A Review of the Impact and Effectiveness of the Quadrivalent Human Papillomavirus Vaccine: 10 Years of Clinical Experience in Canada” | Systematic Review | Canada | N/A | 4vHPV | N/A | N/A | N/A | HPV infection, HPV associated anogenital warts, and/or HPV-associated cervical dysplasia or cervical intraepithelial neoplasia | 7 articles |
|  | Spinner et al.(19) | 2019 | “Human Papillomavirus Vaccine Effectiveness and Herd Protection in Young Women” | Surveillance study | US | N/A | 4vHPV  9vHPV | N/A | N/A | N/A | 1) Prevalence of vaccine type HPV in adolescent and young adult women who were vaccinated (to assess vaccine effectiveness)  2) Prevalence of vaccine-type HPV in women who were unvaccinated (to assess herd protection) | 1580 |
| Durability | Artemchuk et al.(20) | 2019 | Finnish Maternity Cohort biobank | Cohort | Finland | Pregnant women who received HPV vaccine | 4vHPV 2vHPV | 4v: 3 (0, 2, 6) 2v: (0, 1, 6) | Age matched unvaccinated, HPV sero positive women | None | Immune response durability | 4vHPV (n=79)  2vHPV (n=11)  Unvaccinated (n=125) |
|  | Guevara et al.(21) | 2017 | “Antibody persistence and evidence of immune memory at 5years following administration of the 9-valent HPV vaccine” | Cohort | Europe and Latin America | 17-26yo women at vaccination | 9vHPV | 3 (0, 2,6) | N/A | N/A | Immune response durability | 150 |
|  | Kjaer et al.(22) | 2020 | FUTURE II | Cohort | Nordic countries | 16-23 at enrollment | 4vHPV | 3 (0, 2,6) | N/A | N/A | HPV 16/18 related CIN2+ | 2,121 |
|  | Kreimer et al.(23) | 2015 | CVT and PATRICIA | Cohort | Costa Rica | 15-25yo women | 2vHPV | 3 (0, 1, 6) | Hep A vaccine | 3 (0, 1,6) | Persistent HPV, CIN2+ | 22,327  2vHPV (n=11,104)  Hep A Vaccine (n=11,209) |
|  | Safaeian et al.(24) | 2018 | CVT | Cohort | Costa Rica | 18-25yo women | 2vHPV | 3 (0, 1, 6) | N/A | N/A | Persistent HPV, CIN2+ | 2vHPV (n=2043) |
|  | Tsang et al.(25) | 2020 | CVT | Cohort | Costa Rica | 18-25yo women | 2vHPV | 3 (0, 1, 6) | Unvaccinated women | N/A | HPV 31/33/45 infection | At the final visit in the 9-11 year loss to follow-up cohort  3-doses (n=2,102)  Control (n=2,379) |
| Coverage | Bruni et al.(26) | 2016 | “Global estimates of human papillomavirus vaccination coverage by region and income level: a pooled analysis” | Systematic review | Global | N/A | Any HPV vaccines | N/A | N/A | N/A | HPV vaccine coverage | N/A |
|  | Gallagher et al.(27) | 2017 | “Human papillomavirus (HPV) vaccine coverage achievements in low and middle-income countries 2007-2016” | Descriptive analysis | 59 LMICs | N/A | Any HPV vaccines | N/A | N/A | N/A | HPV vaccine coverage | 6 national programs 48 demo project |

**References**

1. Huh WK, Joura EA, Giuliano AR, Iversen OE, de Andrade RP, Ault KA, et al. Final efficacy, immunogenicity, and safety analyses of a nine-valent human papillomavirus vaccine in women aged 16-26 years: a randomised, double-blind trial. Lancet (London, England). 2017;390(10108):2143-59.

2. Paavonen J, Naud P, Salmerón J, Wheeler C, Chow SN, Apter D, et al. Efficacy of human papillomavirus (HPV)-16/18 AS04-adjuvanted vaccine against cervical infection and precancer caused by oncogenic HPV types (PATRICIA): final analysis of a double-blind, randomised study in young women. The Lancet. 2009;374(9686):301-14.

3. Apter D, Wheeler CM, Paavonen J, Castellsague X, Garland SM, Skinner SR, et al. Efficacy of human papillomavirus 16 and 18 (HPV-16/18) AS04-adjuvanted vaccine against cervical infection and precancer in young women: final event-driven analysis of the randomized, double-blind PATRICIA trial. Clinical and vaccine immunology : CVI. 2015;22(4):361-73.

4. Garland SM, Hernandez-Avila M, Wheeler CM, Perez G, Harper DM, Leodolter S, et al. Quadrivalent vaccine against human papillomavirus to prevent anogenital diseases. N Engl J Med. 2007;356(19):1928-43.

5. FUTURE II Study Group. Quadrivalent vaccine against human papillomavirus to prevent high-grade cervical lesions. N Engl J Med. 2007;356(19):1915-27.

6. Kahn JA, Xu J, Kapogiannis BG, Rudy B, Gonin R, Liu N, et al. Immunogenicity and safety of the human papillomavirus 6, 11, 16, 18 vaccine in HIV-infected young women. Clinical infectious diseases : an official publication of the Infectious Diseases Society of America. 2013;57(5):735-44.

7. Kojic EM, Kang M, Cespedes MS, Umbleja T, Godfrey C, Allen RT, et al. Immunogenicity and safety of the quadrivalent human papillomavirus vaccine in HIV-1-infected women. Clinical infectious diseases : an official publication of the Infectious Diseases Society of America. 2014;59(1):127-35.

8. Giacomet V, Penagini F, Trabattoni D, Vigano A, Rainone V, Bernazzani G, et al. Safety and immunogenicity of a quadrivalent human papillomavirus vaccine in HIV-infected and HIV-negative adolescents and young adults. Vaccine. 2014;32(43):5657-61.

9. McClymont E, Lee M, Raboud J, Coutlee F, Walmsley S, Lipsky N, et al. The Efficacy of the Quadrivalent Human Papillomavirus Vaccine in Girls and Women Living With Human Immunodeficiency Virus. Clinical infectious diseases : an official publication of the Infectious Diseases Society of America. 2019;68(5):788-94.

10. Mugo NR, Eckert L, Magaret AS, Cheng A, Mwaniki L, Ngure K, et al. Quadrivalent HPV vaccine in HIV-1-infected early adolescent girls and boys in Kenya: Month 7 and 12 post vaccine immunogenicity and correlation with immune status. Vaccine. 2018;36(46):7025-32.

11. Levin MJ, Moscicki AB, Song LY, Fenton T, Meyer WA, 3rd, Read JS, et al. Safety and immunogenicity of a quadrivalent human papillomavirus (types 6, 11, 16, and 18) vaccine in HIV-infected children 7 to 12 years old. Journal of acquired immune deficiency syndromes (1999). 2010;55(2):197-204.

12. Weinberg A, Song LY, Saah A, Brown M, Moscicki AB, Meyer WA, 3rd, et al. Humoral, mucosal, and cell-mediated immunity against vaccine and nonvaccine genotypes after administration of quadrivalent human papillomavirus vaccine to HIV-infected children. J Infect Dis. 2012;206(8):1309-18.

13. Toft L, Storgaard M, Muller M, Sehr P, Bonde J, Tolstrup M, et al. Comparison of the immunogenicity and reactogenicity of Cervarix and Gardasil human papillomavirus vaccines in HIV-infected adults: a randomized, double-blind clinical trial. J Infect Dis. 2014;209(8):1165-73.

14. Faust H, Toft L, Sehr P, Muller M, Bonde J, Forslund O, et al. Human Papillomavirus neutralizing and cross-reactive antibodies induced in HIV-positive subjects after vaccination with quadrivalent and bivalent HPV vaccines. Vaccine. 2016;34(13):1559-65.

15. Arbyn M, Xu L. Efficacy and safety of prophylactic HPV vaccines. A Cochrane review of randomized trials. Expert Rev Vaccines. 2018;17(12):1085-91.

16. Signorelli C, Odone A, Ciorba V, Cella P, Audisio RA, Lombardi A, et al. Human papillomavirus 9-valent vaccine for cancer prevention: a systematic review of the available evidence. Epidemiol Infect. 2017;145(10):1962-82.

17. Patel C, Brotherton JM, Pillsbury A, Jayasinghe S, Donovan B, Macartney K, et al. The impact of 10 years of human papillomavirus (HPV) vaccination in Australia: what additional disease burden will a nonavalent vaccine prevent? Euro surveillance : bulletin Europeen sur les maladies transmissibles = European communicable disease bulletin. 2018;23(41).

18. Steben M, Tan Thompson M, Rodier C, Mallette N, Racovitan V, DeAngelis F, et al. A Review of the Impact and Effectiveness of the Quadrivalent Human Papillomavirus Vaccine: 10 Years of Clinical Experience in Canada. Journal of Obstetrics and Gynaecology Canada. 2018;40(12):1635-45.

19. Spinner C, Ding L, Bernstein DI, Brown DR, Franco EL, Covert C, et al. Human Papillomavirus Vaccine Effectiveness and Herd Protection in Young Women. Pediatrics. 2019;143(2).

20. Artemchuk H, Eriksson T, Poljak M, Surcel HM, Dillner J, Lehtinen M, et al. Long-term Antibody Response to Human Papillomavirus Vaccines: Up to 12 Years of Follow-up in the Finnish Maternity Cohort. J Infect Dis. 2019;219(4):582-9.

21. Guevara A, Cabello R, Woelber L, Moreira ED, Jr., Joura E, Reich O, et al. Antibody persistence and evidence of immune memory at 5years following administration of the 9-valent HPV vaccine. Vaccine. 2017;35(37):5050-7.

22. Kjaer SK, Nygård M, Sundström K, Dillner J, Tryggvadottir L, Munk C, et al. Final analysis of a 14-year long-term follow-up study of the effectiveness and immunogenicity of the quadrivalent human papillomavirus vaccine in women from four nordic countries. EClinicalMedicine. 2020;23:100401.

23. Kreimer AR, Struyf F, Del Rosario-Raymundo MR, Hildesheim A, Skinner SR, Wacholder S, et al. Efficacy of fewer than three doses of an HPV-16/18 AS04-adjuvanted vaccine: combined analysis of data from the Costa Rica Vaccine and PATRICIA Trials. Lancet Oncol. 2015;16(7):775-86.

24. Safaeian M, Sampson JN, Pan Y, Porras C, Kemp TJ, Herrero R, et al. Durability of Protection Afforded by Fewer Doses of the HPV16/18 Vaccine: The CVT Trial. JNCI: Journal of the National Cancer Institute. 2017;110(2):205-12.

25. Tsang SH, Sampson JN, Schussler J, Porras C, Wagner S, Boland J, et al. Durability of Cross-Protection by Different Schedules of the Bivalent HPV Vaccine: The CVT Trial. Journal of the National Cancer Institute. 2020;112(10):1030-7.

26. Bruni L, Diaz M, Barrionuevo-Rosas L, Herrero R, Bray F, Bosch FX, et al. Global estimates of human papillomavirus vaccination coverage by region and income level: a pooled analysis. The Lancet Global health. 2016;4(7):e453-63.

27. Gallagher KE, Howard N, Kabakama S, Mounier-Jack S, Burchett HED, LaMontagne DS, et al. Human papillomavirus (HPV) vaccine coverage achievements in low and middle-income countries 2007-2016. Papillomavirus Res. 2017;4:72-8.
